# A novel machine learning-based screening identifies statins as inhibitors of the calcium pump SERCA

**DOI:** 10.1016/j.jbc.2023.104681

**Published:** 2023-04-06

**Authors:** Carlos Cruz-Cortés, M. Andrés Velasco-Saavedra, Eli Fernández-de Gortari, Guadalupe Guerrero-Serna, Rodrigo Aguayo-Ortiz, L. Michel Espinoza-Fonseca

**Affiliations:** 1 Division of Cardiovascular Medicine, Department of Internal Medicine, Center for Arrhythmia Research, University of Michigan, Ann Arbor, Michigan, USA; 2 Departamento de Farmacia, Facultad de Química, Universidad Nacional Autónoma de México, Mexico City, Mexico; 3 Department of Nanosafety, International Iberian Nanotechnology Laboratory, Braga, Portugal

**Keywords:** machine learning, calcium ATPase, drug screening, molecular pharmacology, membrane protein, inhibition mechanism, statins, SERCA, data augmentation

## Abstract

We report a novel small-molecule screening approach that combines data augmentation and machine learning to identify Food and Drug Administration (FDA)-approved drugs interacting with the calcium pump (Sarcoplasmic reticulum Ca^2+^-ATPase, SERCA) from skeletal (SERCA1a) and cardiac (SERCA2a) muscle. This approach uses information about small-molecule effectors to map and probe the chemical space of pharmacological targets, thus allowing to screen with high precision large databases of small molecules, including approved and investigational drugs. We chose SERCA because it plays a major role in the excitation-contraction-relaxation cycle in muscle and it represents a major target in both skeletal and cardiac muscle. The machine learning model predicted that SERCA1a and SERCA2a are pharmacological targets for seven statins, a group of FDA-approved 3-hydroxy-3-methylglutaryl coenzyme A reductase inhibitors used in the clinic as lipid-lowering medications. We validated the machine learning predictions by using *in vitro* ATPase assays to show that several FDA-approved statins are partial inhibitors of SERCA1a and SERCA2a. Complementary atomistic simulations predict that these drugs bind to two different allosteric sites of the pump. Our findings suggest that SERCA-mediated Ca^2+^ transport may be targeted by some statins (*e.g.*, atorvastatin), thus providing a molecular pathway to explain statin-associated toxicity reported in the literature. These studies show the applicability of data augmentation and machine learning-based screening as a general platform for the identification of off-target interactions and the applicability of this approach extends to drug discovery.

Machine learning, a branch of artificial intelligence, is making a mark in the pharmaceutical industry, contributing to exciting innovations in drug discovery (1). Machine learning provides unprecedentedly rich information that can extract underlying patterns and build predictive models of complex data (2). There is an urgent need for the identification of off-target effects and mechanisms for approved and investigational drugs (3). However, screening campaigns are time-consuming and expensive (4), and there are currently no systematic, rapid, and low-cost platforms for the identification of off-target pathways and underlying molecular mechanisms for approved and investigational drugs. Here, we propose a machine learning approach that uses an objective function allowing the inclusion of chemical space enrichment (5). In conjunction with this method, we used data augmentation to alleviate data scarcity scenarios (*e.g.*, a limited dataset of effector molecules) where machine learning techniques may fail. This combined approach enables classifiers to confine a region of the chemical space into bioactive molecules to effectively characterize the pharmacological space of a target of interest. This pharmacological space can then be probed to screen for drugs that match the pharmacological space of the target(s). We complement this approach with functional assays and atomistic molecular simulations to validate and complement the machine learning predictions.

In this study, we used machine learning-based screening to investigate the inhibition of the calcium pump (sarcoplasmic reticulum [SR] Ca^2+^-ATPase, [SERCA]) by Food and Drug Administration (FDA)-approved drugs. The structure of SERCA is highly conserved, exhibiting variations only in the extension of the C-terminal region across isoforms and splice variants. Nine splice variants of SERCA (SERCA1a-b, SERCA2a-d, SERCA3a-c) encoded by three different genes (ATP2A1-3) have been identified in humans (6). We focus on two variants of the Ca^2+^ pump: SERCA1a, primarily expressed in skeletal muscle, and SERCA2a, predominantly found in cardiac muscle (7). In muscle, SERCA1a and SERCA2a play a central role in Ca^2+^ homeostasis and contractility, as they establish vital Ca^2+^ gradients across the SR respectively (8). SERCA is a key pharmacological target both for drug development and as potential proteins for off-target effects since they play major roles in normal and pathological conditions (9).

We tested a novel approach for data augmentation of the original training dataset composed of SERCA inhibitors to create robust maps for the pharmacological space of SERCA. We used this approach to show that SERCA1a and SERCA2a are inhibited by statins, a group of FDA-approved inhibitors of the 3-hydroxy-3-methylglutaryl CoA reductase (HMG-CoA), which are used as lipid-lowering medications (10, 11, 12). We used *in situ* enzymatic assays to show that several statins are inhibitors of SERCA1a and SERCA2a. Complementary atomistic simulations predict that these drugs bind to two different effector sites of the pump. Our studies showed that the statin atorvastatin lactone is a partial micromolar inhibitor of SERCA1a and SERCA2a. The potency of this statin correlates with a higher incidence of statin toxicity reported in the literature (13). These findings support the use of data augmentation to improve the predictive power of machine learning-based drug screening for approved and investigational drugs.

## Results

### Data augmentation improves the performance of the classifier for SERCA inhibitors

In many real-world application settings, such as drug discovery, a problem often encountered is having sufficient training data for machine learning applications and data augmentation is currently the most effective way of overcoming this problem. The main goal of data augmentation is to increase the volume, quality, and diversity of training data. In our study, we take advantage of this and use a conservative approach to data augmentation assuming a continuous structure-activity relationships (SAR) model around known inhibitors to train our model, as described below.

We first trained the SERCA inhibitors classifier model using the original dataset of nonredundant SERCA inhibitors and inactive molecules (decoys) curated from the PubChem databank (14). We used different methodological strategies and data process variations to avoid model overfitting because of the limited number of known SERCA inhibitors and inactive molecules. These strategies included data representation by different approaches, including latent space embeddings, extended connectivity fingerprints, pharmacophore-based fingerprints, and dictionary-based fingerprints (15). Initial training included grid search for model selection (Random Forest, Support Vector Machine, Logistic regression, and k-nearest neighbors), and 5-fold cross-validation for hyperparameters tuning to determine the model performance by comparing correlation metrics between the training and test set metrics of the original sets of small molecules.

**Figure 1:**
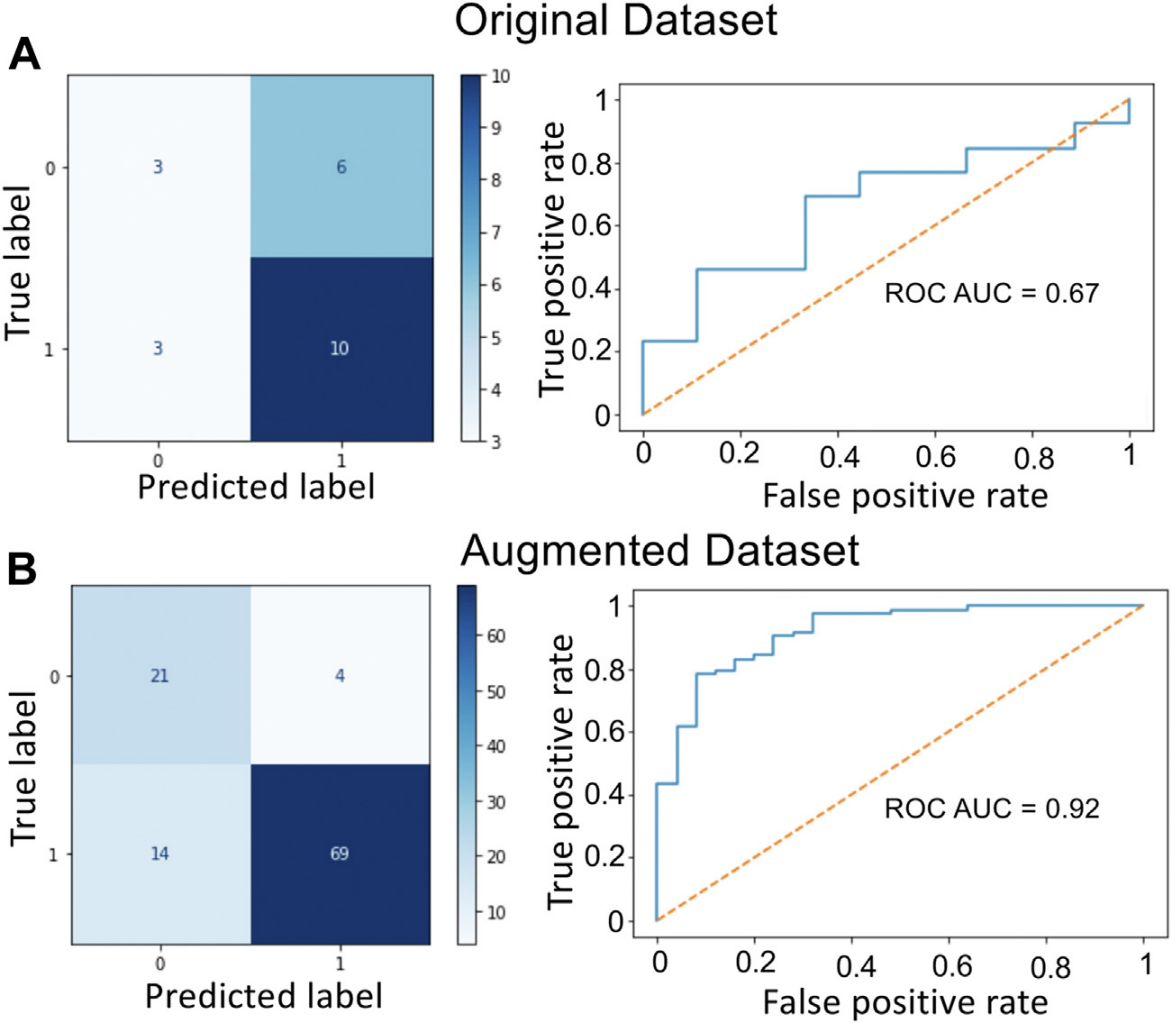
**Performance of the machine learning model using original and augmented datasets of SERCA inhibitors.** We evaluated the performance of the machine learning models by using the confusion matrix, a widely used summary of prediction results of a classification problem applied to binary classification as well as for multiclass classification problems. Confusion matrices represent counts from predicted and actual (true) values. *A*, confusion matrix and ROC curve of the model using the original dataset of SERCA inhibitors. *B*, confusion matrix and ROC curve of the model using the augmented dataset of SERCA inhibitors. In all cases, a value of one for both true and predicted labels is assigned to SERCA inhibitors, whereas a value of 0 is assigned to inactive molecules. ROC, receiver-operator curve; SERCA, sarcoplasmic reticulum Ca^2+^-ATPase.

We found that, when applied to the original set of SERCA inhibitors and inactive molecules, all the above strategies produced overfitted models. For instance, the confusion matrix of the original inhibitor dataset revealed that applying the Random Forest approach with the 5-fold cross-validation using the dataset of published SERCA inhibitors produces near-perfect performance when applied to the training set but the performance gap is large when applied to a test set (Fig. 1*A* and Table 1). In this case, the model trained with the original dataset cannot generalize and fits too closely to the training dataset. To overcome this problem, we performed data augmentation using a generative approach capable of breeding new structures starting from a seed compound by growing, mutating, or linking a predefined set of fragments that comply with the seed molecule. By using this approach, we can ensure that the rate of activity cliffs (*i.e.*, loss-of-function) is low and a continuous SAR of the generated molecules. We, therefore, generated molecular datasets by growing the original compounds in the training set, active and inactive class, with a higher level of conservatism which maintains the original characteristics required for SERCA inhibition (Fig. S1, Supporting Information).

**Table 1:** Summary of performance of the original dataset of SERCA inhibitors

Set type	Accuracy^a^	Matthews^a^	F1-score^a^
Training	1.0	1.0	1.0
Test	0.59	0.11	0.57
Δ(Training - Test)	0.41	0.89	0.43

Abbreviation: SERCA, sarcoplasmic reticulum Ca^2+^-ATPase.

a We use a scale of 0 to 1, where 0 and 1 indicates poor and perfect predictive performance predictive capabilities of the model on a given set (training or test).

The augmented dataset, which is 20 times larger than the original dataset of SERCA inhibitors and inactive molecules, was used to train the previously selected model as a starting point for 10-fold cross-validation and grid-search for the new model hyperparameter tuning. In comparison with the model trained with the original dataset, the augmented dataset improves the performance of the classifier, illustrated by a narrow gap in the performance and accuracy between the training and test sets (Fig. 1*B* and Table 2). The result is a continuous map representing the space occupied by SERCA inhibitors and inactive molecules (Fig. S1, Supporting Information). These results demonstrate that, in the absence of large datasets, the expansion of the chemical space proposed here recapitulates the fundamental chemical properties that are required for SERCA inhibition. These findings also demonstrate that the performance of the machine learning model is considerably better in combination with data augmentation than with the original compound dataset alone.

**Table 2:** Summary of performance of the augmented dataset of SERCA inhibitors

Set type	Accuracy^a^	Matthews^a^	F1-score^a^
Training	0.87	0.78	0.90
Test	0.84	0.60	0.84
Δ(Training - Test)	0.03	0.18	0.06

Abbreviation: SERCA, sarcoplasmic reticulum Ca^2+^-ATPase.

a We use a scale of 0 to 1, where 0 and 1 indicates poor and perfect predictive performance predictive capabilities of the model on a given set (training or test).

### Machine learning with an augmented dataset predicts FDA-approved drugs as inhibitors of SERCA

We used the best machine learning model trained here to screen for 1447 FDA-approved drugs as inhibitors of SERCA. Based on our recent studies (5), we selected drugs with class probability values between 0.6 and 0.9 and neighbor count larger than three as initial filtering parameters to determine whether SERCA is an off-target for approved drugs. We applied a dimensionality reduction and visualization for the resulting molecules using the T-distributed stochastic neighbor embedding (t-SNE) (16). t-SNE is a nonlinear dimensionality reduction technique used for embedding high-dimensional data (*e.g.*, chemical space) in a low-dimensional space of two or three dimensions. We also used the number of hydrogen-bond donors and acceptors, the partition coefficient, and the molecular weight of small molecules as additional filters to further restrict the space to that occupied by known SERCA inhibitors.

**Figure 2:**
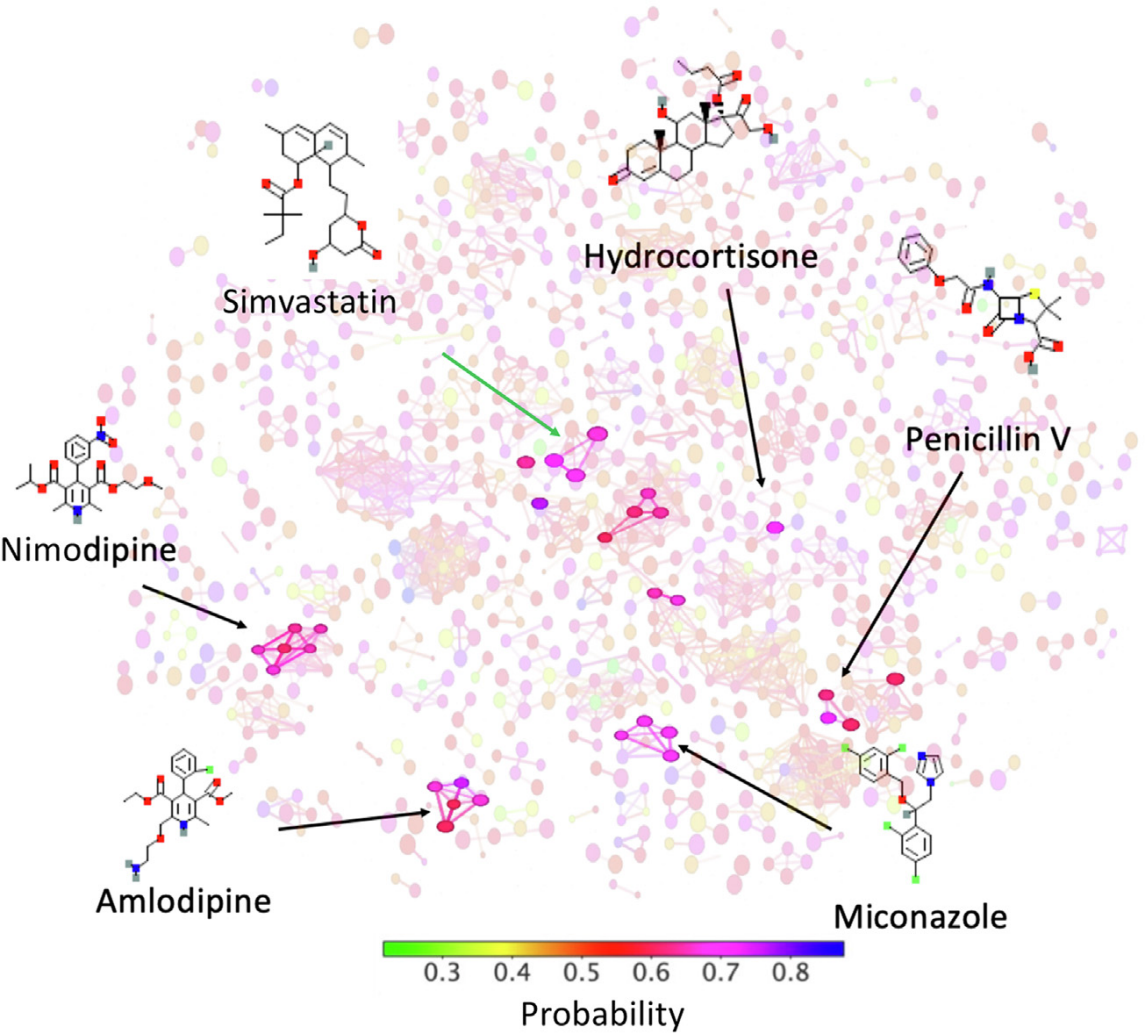
**A t-SNE dimensionality reduction and network representation of the FDA-approved drugs.** We used t-SNE as a technique for nonlinear dimensionality reduction used for embedding the high-dimensional chemical space of SERCA inhibition in a low-dimensional space. Drugs are represented as nodes linked by their structural similarity. Each node is color-coded by the output probability calculated by the classifier developed using the augmented dataset of SERCA inhibitors. Upon application of filters, the machine learning model predicted that FDA-approved drugs simvastatin, nimodipine, amlodipine, hydrocortisone, penicillin V, and miconazole are SERCA inhibitors. We show the structures and localization in the network of the most representative drugs predicted by the machine learning model. FDA, Food and Drug Administration; t-SNE, T-distributed stochastic neighbor embedding.

**Figure 3:**
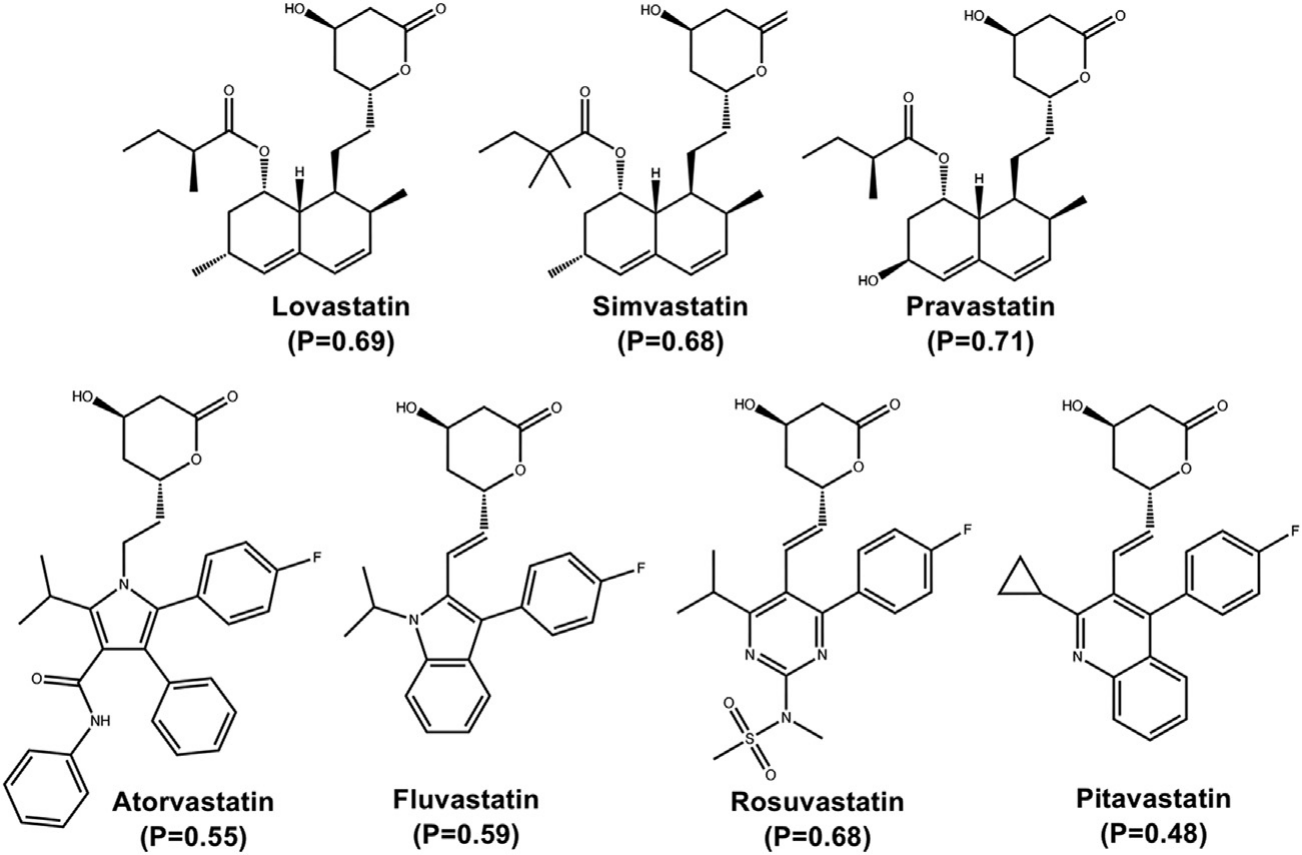
**Chemical structures of FDA-approved statins.** The predicted probability for each statin as SERCA inhibitors is shown in parenthesis. The *upper row* shows chemically related lovastatin, simvastatin, and pravastatin, which are characterized by the presence of a naphthalene ring and a lactone group. The *lower row* shows the chemical structures of atorvastatin, fluvastatin, rosuvastatin, and pitavastatin, characterized by either a pyrrole or an indole group and a lactone moiety. All seven statins are shown in their lactone form. FDA, Food and Drug Administration; SERCA, sarcoplasmic reticulum Ca^2+^-ATPase.

The 2-dimensional t-SNE mapping approach showed a dense cluster of molecules with consistent probability values of 0.6 to 0.9, which we interpret as a conservation of the pharmacophoric elements representing inhibition of SERCA (Fig. 2). After applying the molecular filters described above, our model predicts several FDA-approved drugs as SERCA inhibitors, including the cholesterol-lowering drug simvastatin, the calcium channel blockers amlodipine and nimodipine, hydrocortisone, which is used for adrenocortical insufficiency, the antibiotic penicillin V, and the antimycotic miconazole (Fig. 2). Interestingly, the machine learning model predicts that seven FDA-approved statins are inhibitors of SERCA (Fig. 3). Statins are a group HMG-CoA reductase inhibitors indicated for the primary and secondary prevention of atherosclerotic cardiovascular events (17).

Inhibition of SERCA by statins, as predicted by our machine learning model, is significant because previous studies have shown that these drugs stimulate the activity of SERCA in skeletal and vascular muscle (18, 19) (in contrast to our predictions) and because statin toxicity is welldocumented (13), and includes statin-associated muscle symptoms (SAMS) (20). There are also studies in rat skeletal muscles (21), human skeletal muscle cells (22), and human-derived biopsies (23) showing that mitochondria and Ca^2+^ homeostasis are disturbed following the acute application of statins. These facts agree with some other reports which suggest that statin-related myopathies had the origin of these alterations in Ca^2+^ homeostasis (23, 24, 25). Based on these studies and our own model, we hypothesize that statins exert some of these effects by inhibiting SERCA in the muscle. Consequently, we tested the inhibition of SERCA1a and SERCA2a by statins *in vitro*.

### Inhibition of SERCA1a and SERCA2a by lovastatin, simvastatin, and pravastatin

Lovastatin, pravastatin, and simvastatin are structurally similar, containing a naphthalene ring and a lactone group (Fig. 3). These statins exist as either a lipophilic lactone ring or as an hydroxyglutaric acid (26). Therefore, both statin lactone and statin acid were tested for ATPase activity using both skeletal muscle SERCA1a and cardiac muscle SERCA2a. In all cases, activity is represented as the % of the change in V_max_ relative to the activity of untreated SERCA (negative control). A summary of the parameters derived from the concentration-response fitting model is shown in Tables S1 and S2. When describing the data, we make a distinction between “observed” data (*i.e.*, datapoints) and “estimated” data (*i.e.*, values derived from the fitted model data).

**Figure 4:**
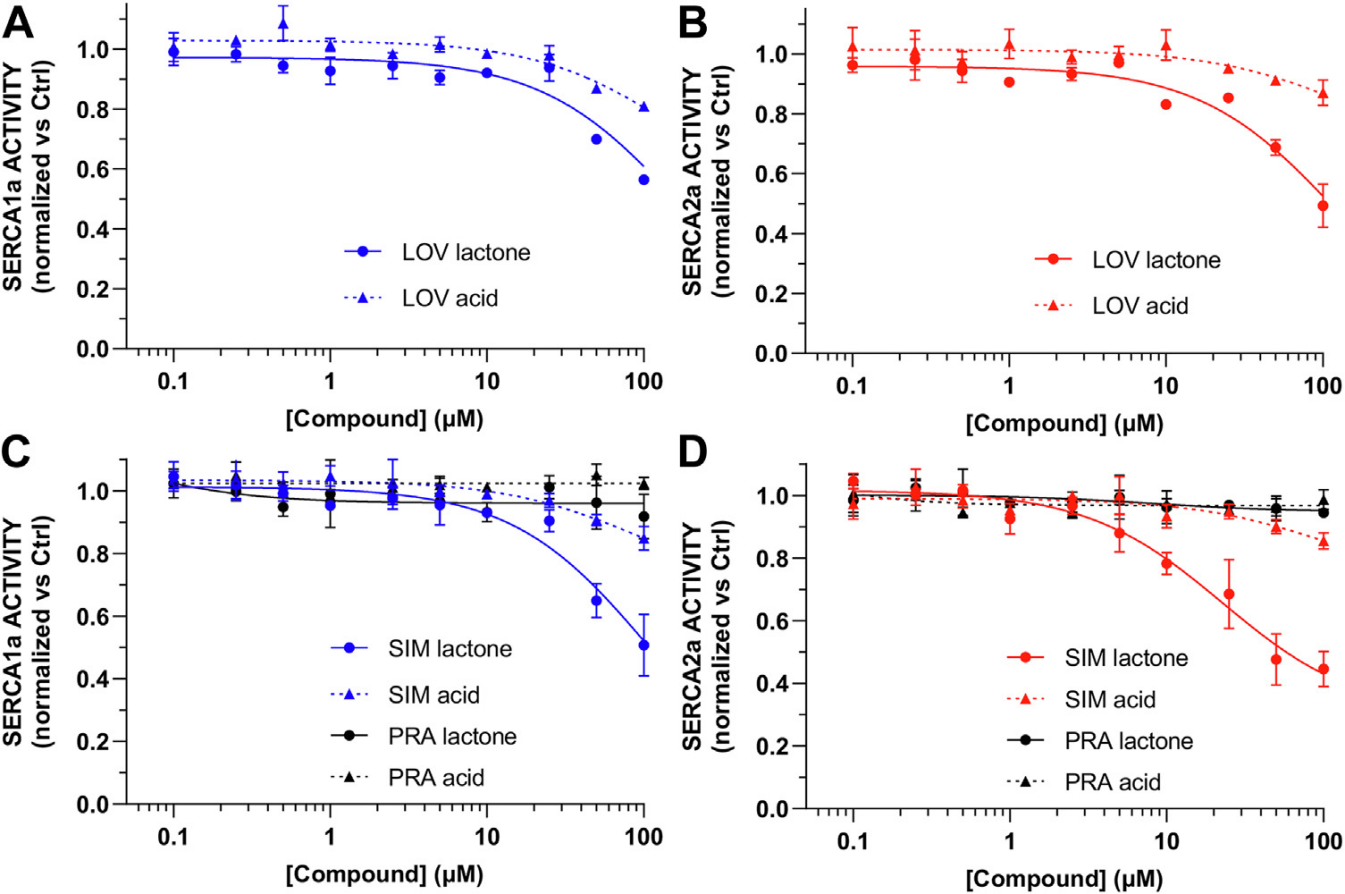
**Effects of lovastatin, simvastatin, and pravastatin on SERCA1 and SERCA2a.** Ten-point concentration-response curves of lovastatin tested against (*A*) skeletal muscle SERCA1a and (*B*) cardiac SERCA2a. The activity of simvastatin and pravastatin was tested against (*C*) SERCA1a and (*D*) SERCA2a. In all cases, the activity of the compounds at each concentration was obtained from 2.5 μM free [Ca^2+^]-dependent SERCA activity and normalized relative to the negative control as described in Experimental procedures. In all plots, *solid* and *dashed traces* show the activity of a given statin in its lactone and acid form, respectively. Data are reported as average ± SD (N = 3). SERCA, sarcoplasmic reticulum Ca^2+^-ATPase.

Lovastatin had a moderate inhibitory effect on the activity of SERCA1a, with an observed inhibition of SERCA activity by 44 ± 1% and 19 ± 2% at 100 μM for the lactone and acid forms, respectively (Fig. 4*A*). Similarly, lovastatin lactone decreased the activity of SERCA2a by 51 ± 7%, whereas the acid form inhibited this isoform only by a 13 ± 4% observed at the highest concentration of the compounds tested here (Fig. 4*B*). Based on the fitted concentration-response curves, simvastatin lactone inhibits both SERCA1a and SERCA2a (Fig. 4*C*). Nonetheless, simvastatin lactone has a more pronounced inhibitory effect on SERCA2a, with an IC_50_ value of 22.7 ± 6.1 μM and a maximal inhibition of 70 ± 6% calculated from the fitted model (Table S2). Like lovastatin, the activity of the acid form of simvastatin is substantially less inhibitory that its lactone counterpart (Fig. 4*C*, Tables S2 and S3).

Pravastatin, which has the highest predicted class probability among the statins identified by our model, had no inhibitory effect on SERCA1a or SERCA2a either in its lactone or acid form (Fig. 4, *C* and *D*). These findings are corroborated by a poor fit of the data to a concentration-response curve (R^2^ < 0.18, Tables S1 and S2). These findings are surprising because pravastatin is chemically identical to the active molecule simvastatin, except for the hydroxyl substitution in the pravastatin molecule (Fig. 3). We, therefore, used complementary docking, molecular dynamics (MD) simulations, and membrane permeability predictions to explain the loss of inhibitory activity induced by the hydroxyl group of pravastatin.

**Figure 5:**
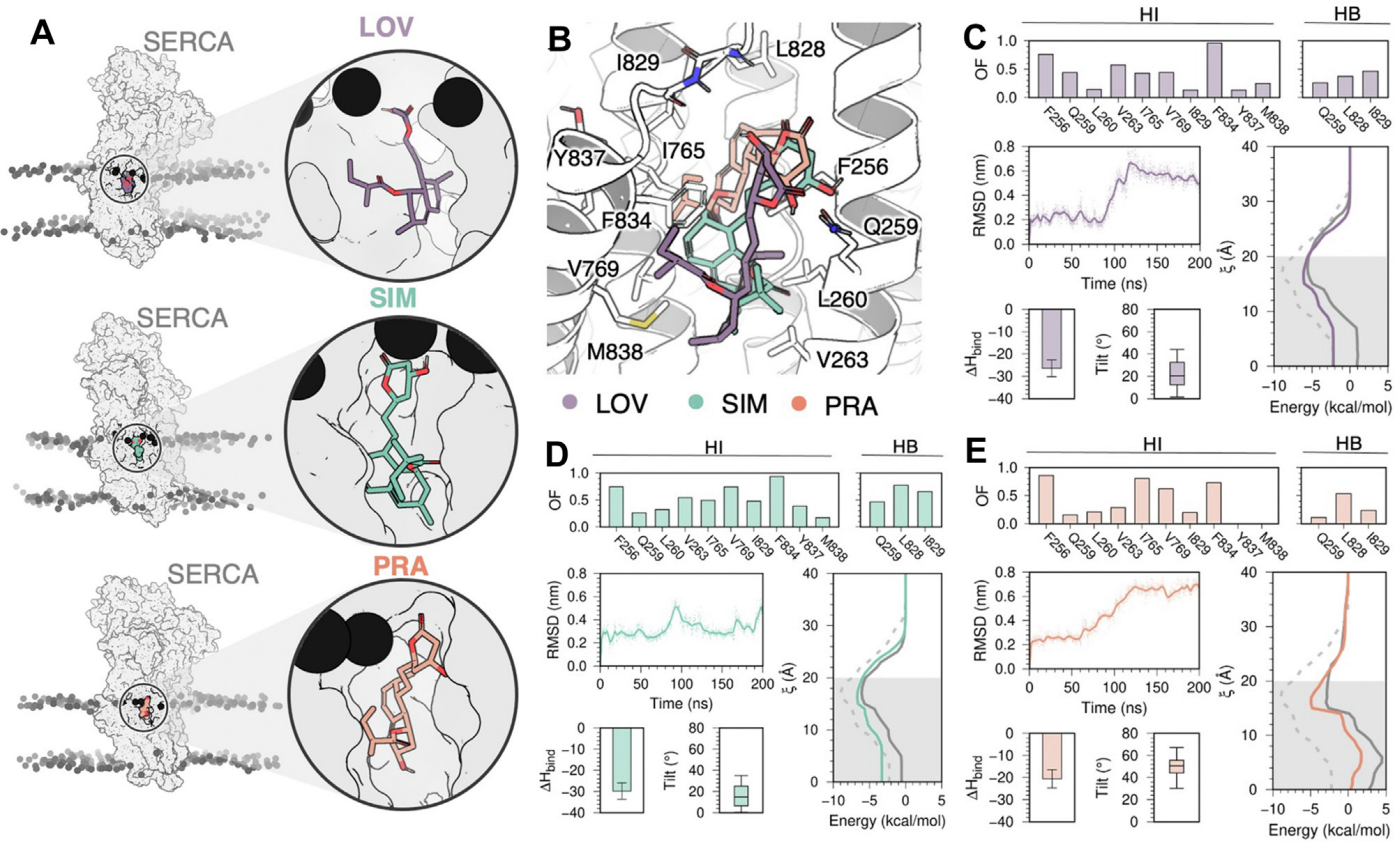
**Predicted SERCA-statin interactions and passive diffusion profiles of lovastatin, simvastatin, and pravastatin.***A*, extensive docking simulations using 85 crystal structures of SERCA representing all major intermediate states of the pump predict that these three statins bind to the TG-binding site located in the transmembrane domain of SERCA. The most representative binding orientation of lovastatin, simvastatin, and pravastatin predicted by the docking engine are shown in circles. Statins are shown as sticks and SERCA as a surface representation. The boundaries of the lipid bilayer are shown as spheres. *B*, orientation of all three statins in the TG-binding site predicted by docking simulations. Statins are shown as sticks and SERCA as ribbons. Hydrophobic (HI) and hydrogen-bond (HB) occupancy frequency plots, time-dependent RMSD, enthalpy of binding (ΔH_bind_), tilt angle, and passive diffusion profiles obtained for (*C*) lovastatin, (*D*) simvastatin, and (*E*) pravastatin. The traces in the passive diffusion profiles are as follows: *solid-colored* and *gray traces* show the profiles for the lactone and acid statins, respectively; the *dashed trace* is the profile for the control compound thapsigargin. SERCA, sarcoplasmic reticulum Ca^2+^-ATPase.

We used molecular docking simulations previously validated against known crystallographic structures (Table S3). These studies predict that lovastatin, pravastatin, and simvastatin in their lactone forms preferably bind to the E2 state of the pump (Tables S4 and S5, Supporting Information). The predictions show that these statins bind to the thapsigargin (TG)-binding site, which is found in the cytosolic side of the transmembrane domain of the pump (Fig. 5*A*) and is known for playing a central role in allosteric inhibition of the transport cycle (6). Docking studies predicted that lovastatin, simvastatin, and pravastatin molecules in their lactone form adopt a similar orientation on the TG-binding site (Fig. 5*B*). These statins bind to SERCA through interactions with several residues in the TG-binding site, including F256, a critical residue for TG scaffold binding in this site (27).

Upon relaxation of the complex using all-atom MD simulations for 200 ns, lovastatin lactone maintains intermolecular interaction with residues F256, V263, and F834 (Fig. 5*C*), whereas simvastatin lactone interacts with residues F256, V263, I765, V769, I829, and F834 for over 50% of the simulation time (Fig. 5*D*). In the same timescale, residues F256, I765, V769, and F834 interact with pravastatin lactone for >50% of the simulation time (Fig. 5*E*). Analysis of the RMSD plots showed that lovastatin and pravastatin, but not simvastatin, undergo a large shift in their orientation, reflected by RMSD of up to 0.7 nm (Fig. 5, *C* and *D*). The three statin lactones interact favorably with SERCA, as the average enthalpy of binding (ΔH_bind_) is −26 kcal/mol for lovastatin (Fig. 5*C*), −30 kcal/mol for simvastatin (Fig. 5*D*), and −20 kcal/mol for pravastatin (Fig. 5*E*).

The machine learning model and the atomistic simulations predict that lovastatin, simvastatin, and pravastatin interact favorably with SERCA, in contrast with our experimental measurements showing that only lovastatin and simvastatin lactones inhibit SERCA. To explain these differences, we first analyzed the average tilt angle of the molecules as a proxy for the overall orientation of the molecules in the transmembrane TG-binding site. The average tilt angle is 16° and 20° for the functionally active molecules lovastatin and simvastatin (Fig. 5, *C*–*E*) but pravastatin undergoes a 50° change in its tilt angle (Fig. 5*E*). Complementary bilayer-crossing free energy profiles showed that lovastatin and simvastatin lactones have a small membrane entry barrier from the solution and the compounds reach their preferred penetration depth, either close to the lipid headgroup or the membrane midplane (Fig. 5, *C* and *D*). This profile is similar to that of TG (Fig. 5, *C* and *D*, dashed trace), interacting favorably with the lipid bilayer with interaction energies between −5 and −6 kcal/mol. The free-energy profile of lovastatin and simvastatin acid indicates that these compounds interact less favorably with the lipid (Fig. 5, *C* and *D*). Pravastatin in both lactone and acid forms interacts favorably with the lipid headgroup region at around 16 to 20 Å (Fig. 5*E*) but is unable to penetrate deeper into the lipid bilayer as observed for lovastatin, simvastatin, and the control TG.

### Inhibition of SERCA1a and SERCA2a by fluvastatin, atorvastatin, rosuvastatin, and pitavastatin

The second group of statins we tested is composed of atorvastatin, fluvastatin, rosuvastatin, and pitavastatin. This group of statins is structurally more diverse, yet they share either a pyrrole or an indole (a pyrrole ring fused to a phenyl ring) group, and a lactone moiety (Fig. 3). A summary of the parameters derived from the concentration-response fitting model is shown in Tables S1 and S2. When describing the data, we make a distinction between “observed” data (*i.e.*, datapoints) and “estimated” data (*i.e.*, values derived from the fitted model data).

**Figure 6:**
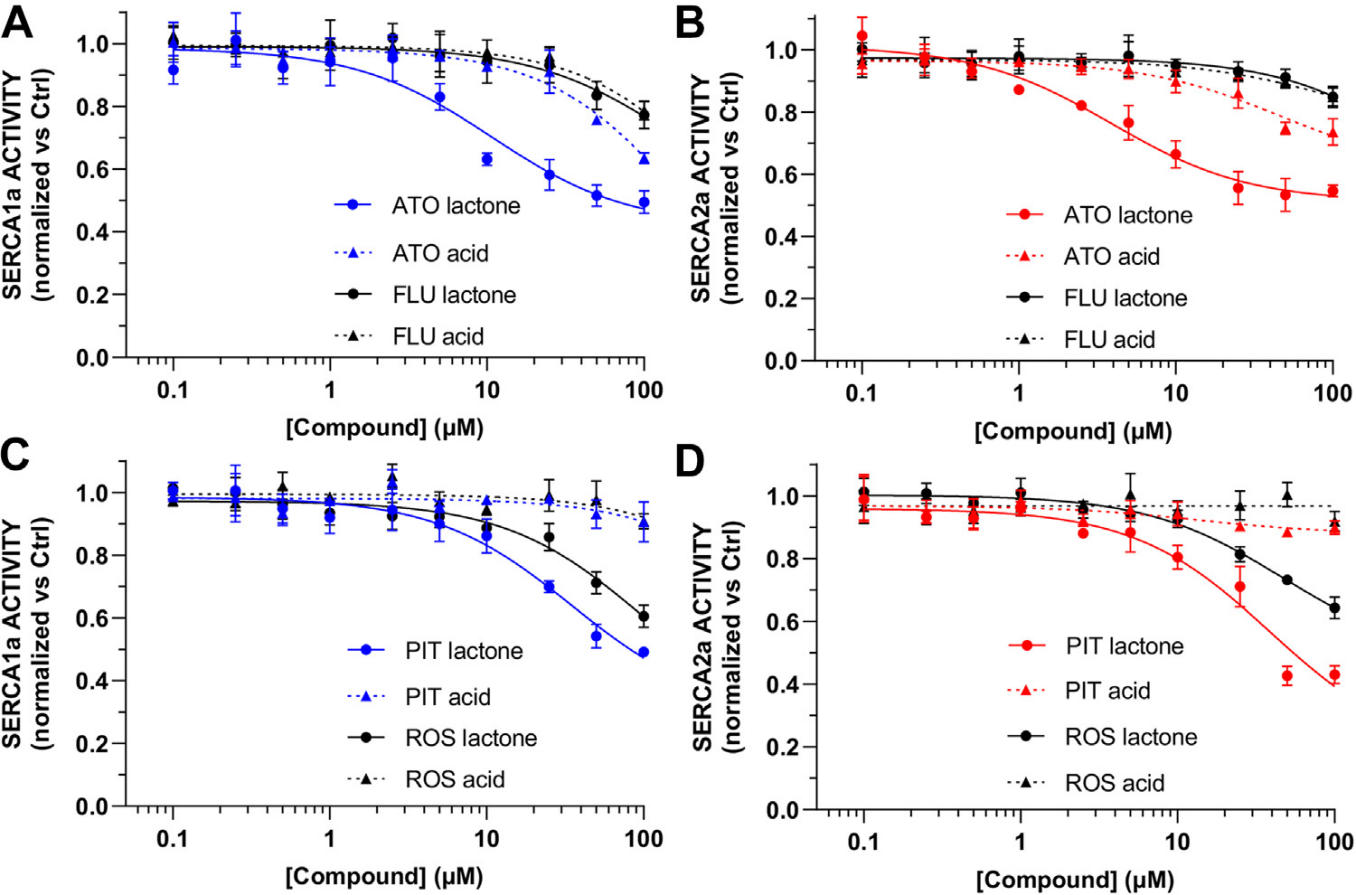
**Effects of atorvastatin, fluvastatin, pitavastatin, and rosuvastatin on SERCA1a and SERCA2a.** Ten-point concentration-response curves of atorvastatin and fluvastatin tested against (*A*) skeletal muscle SERCA1a and (*B*) cardiac SERCA2a. The activity of pitavastatin and rosuvastatin was tested against (*C*) SERCA1a and (*D*) SERCA2a. In all cases, the activity of the compounds at each concentration was obtained from 2.5 μM free [Ca^2+^]-dependent SERCA activity and normalized relative to the negative control as described in Experimental procedures. In all plots, *solid* and *dashed traces* show the activity of a given statin in its lactone and acid form, respectively. Data are reported as average ± SD (N = 3). SERCA, sarcoplasmic reticulum Ca^2+^-ATPase.

Based on the fitted concentration-response curves, atorvastatin lactone is a partial inhibitor of both SERCA isoforms (Fig. 6, *A* and *B*). This compound inactivates SERCA1a with an IC_50_ value of 10.7 ± 3.4 μM and a maximal inhibition of 58 ± 4% calculated from the fitted model (Table S1). Likewise, atorvastatin lactone inhibits SERCA2a with an IC_50_ value of 3.9 ± 0.8 μM and an estimated maximal inhibition of 49 ± 2% (Table S2). Hydrolysis of the lactone ring substantially affects the activity of atorvastatin on SERCA1a but the acid form retains a measurable inhibitory activity against the SERCA2a isoform (Fig. 6, *A* and *B* and Table S2). Fluvastatin, both in its lactone and acid forms, has generally a modest effect on the activity of both SERCA1a and SERCA2a, inhibiting both pumps by 15 to 23% at the maximal compound concentration tested here (observed at 100 μM, Fig. 6, *A* and *B*). Rosuvastatin lactone inhibited both SERCA1a and SERCA2a (Fig. 6, *B* and *C*) with an estimated maximal inhibition of 75 ± 20% and 53 ± 6%, respectively (Tables S1 and S2). However, we found the acid form of rosuvastatin to be inactive against both SERCA isoforms (Fig. 6, *C* and *D*). Finally, pitavastatin lactone is a partial inhibitor of both SERCA isoforms (Fig. 6, *C* and *D*), with a calculated maximal inhibition of 71 ± 8% against SERCA1a, and 83 ± 10% against SERCA2a (Tables S1 and S2). The acid form of pitavastatin has a negligible effect on the activity of both SERCA isoforms (Fig. 6, *C* and *D*).

**Figure 7:**
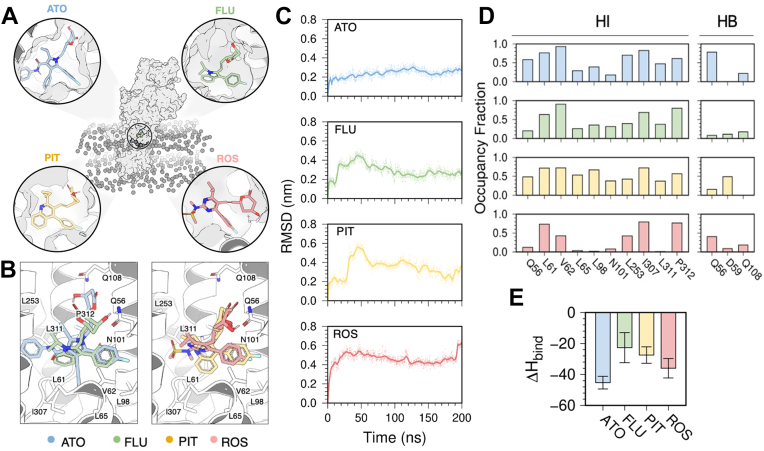
**Predicted Interactions of SERCA with atorvastatin, simvastatin, fluvastatin, and rosuvastatin.***A*, extensive docking simulations using 85 crystal structures of SERCA representing all major intermediate states of the pump predict that these four statins bind to the CPA-binding site located at the lipid-water interface near the cytosolic lipid-water interface of SERCA. The most representative binding orientation of atorvastatin, fluvastatin, pitavastatin, and rosuvastatin predicted by the docking engine are shown in circles. Statins are shown as *sticks* and SERCA as a surface representation. The boundaries of the lipid bilayer are shown as spheres. *B*, orientation of all four statins in the CPA-binding site predicted by docking simulations. Statins are shown as *sticks* and SERCA as *ribbons*. *C*, time-dependent evolution of the RMSD for each statin in the CPA-binding site of the pump. *D*, hydrophobic (HI) and hydrogen-bond (HB) occupancy frequency plots for each statin in the complex with SERCA. *E*, enthalpy of binding (ΔH_bind_) calculated for each statin in the CPA-binding site of SERCA. CPA, cyclopiazonic acid; SERCA, sarcoplasmic reticulum Ca^2+^-ATPase.

We performed complementary docking and MD simulation studies to predict the preferred binding site of fluvastatin, atorvastatin, rosuvastatin, and pitavastatin on SERCA. These statins bind to either E2-P_i_ or E2-P_i_-ATP states of the pump (Tables S4 and S5 in the Supporting Information). In this state of the pump, these statins are predicted to bind to the cyclopiazonic acid (CPA)-binding site of SERCA (Fig. 7, *A* and *B*) (6). This site is near the cytosolic gate of SERCA and binding of inhibitors to this site inhibits SERCA by preventing active site residues of SERCA from adopting an active conformation (6).

MD simulations of the docked structures predict that the statin lactones form stable complexes with SERCA, with atorvastatin lactone being the most stable (average RMSD values ∼0.2 nm) and rosuvastatin lactone being the most mobile (RMSD values of up to 0.6 nm) (Fig. 7*C*). We also modeled and simulated the complex between the statin acids and SERCA to explain the differences in activity observed in the ATPase activation assays. Analysis of the RMSD showed that the position of atorvastatin acid changes by 0.6 nm; the position of fluvastatin acid changes by 2.3 nm; the position of pitavastatin acid is shifted by 0.7 nm; and the position of rosuvastatin acid by 0.4 nm (Figs. S2–S5, Supporting Information). While the differences in RMSD may explain the loss of inhibitory activity of the acid form of these statins, it is also possible that the statin acids bind to SERCA through coordination of the hydroxyglutaric acid moiety through coordination with a negatively charged region near the CPA-binding site (28). We speculate that this factor explains the loss of inhibition observed for the acid form of these statins.

The occupancy fraction plots showed that atorvastatin lactone has the most intermolecular interactions with SERCA, including those with residues L61, V62, L253, I307, and P302. The lactone form of fluvastatin and pitavastatin is stabilized primarily by hydrophobic residues L61, V62, I307, and P312. Rosuvastatin interacts primarily with hydrophobic residues L61, I307, and P312 and to some extent through hydrogen bond interactions with residue Q56 (Fig. 7*D*). Despite these differences, atorvastatin and rosuvastatin in their lactone forms interact favorably with SERCA, (ΔH_bind_ values of −44 and −36 kcal/mol, respectively) followed by pitavastatin (ΔH_bind_ = −28 kcal/mol) and fluvastatin (ΔH_bind_ = −23 kcal/mol) (Fig. 7*E*). Together, these findings show that atorvastatin lactone is predicted to form the most stable complex with SERCA, in agreement with our experiments showing that this is the most potent inhibitory statin among those discovered here.

## Discussion

We report a novel method to construct maps representing the pharmacological space of SERCA and then probe this map to identify approved drugs as inhibitors of this pump. Our approach is innovative as it uses data augmentation in combination with machine learning-based screening as a general platform for drug discovery and identification of off-target interactions.

Machine learning provides unprecedentedly rich information that can extract underlying patterns and build predictive models of complex data. However, a limitation of the use of machine learning methods is the need for “big data”, which is often not readily available given the low throughput of conventional drug discovery campaigns. We overcome this challenge by generating augmented maps representing the pharmacological space for SERCA inhibition. Data augmentation begins with the assumption that given the continuous SAR of the inhibitors' neighborhood, the most probable outcome from a small structural change will be proportional to a change in activity. The small activity difference and the structural similarities across the SERCA inhibitors class reported by SALI (29) support this premise and the expected low probability of activity cliffs (*i.e.*, chemical changes that induce loss of activity) is reported in the chemical space. Therefore, this hypothesis allowed us to apply conservative structural changes to the experimentally tested molecules to generate new consistent molecular structures for data augmentation of the original training dataset. Through this approach, we can set a predefined level of structural conservatism in relation to the initial seed compound, thus allowing us to generate an expanded chemical toolbox (30).

We initially trained the machine learning model using the original dataset of SERCA inhibitors and inactive molecules curated from the PubChem databank (14). We also used this dataset as initial seed structures to expand the chemical space of SERCA inhibition and use this set for training our model. The confusion matrix applied to the original inhibitor dataset revealed that applying the Random Forest approach with the 5-fold cross-validation using the dataset of SERCA inhibitors produces near-perfect performance when applied to the training set but the performance gap is large when applied to a test set. Interestingly, over-fitting of the model is still present after applying standard measures to ease this pitfall, including a reduction in the number of features, feature engineering, transformation and selection, loss-function penalization, model-complexity reduction, and early stopping (data not shown). Collectively, these findings show that, with a limited dataset, the predictive models are prone to poor generalization.

Based on our initial findings, we hypothesized that the reason for the overfitting and poor performance of the classifier is the small number of molecules available for training and testing of the model. To test this hypothesis, we tested a generative approach to generate hypothetical molecules around the original dataset of SERCA inhibitors. By applying this approach, our goal is to produce a continuous chemical space that recapitulates the properties associated with SERCA inhibition. This generative algorithm was applied to the training set, which includes both active and inactive small molecules. The resulting model has better predictive power, illustrated by a narrow gap in the performance and accuracy between the training and test sets. The result is a continuous map representing the space occupied by active and inactive effectors of the SERCA. Through these studies, we demonstrate that in the absence of large datasets, the expansion of the chemical space recapitulates the prototypical elements required for SERCA inhibition, essentially generating maps representing the chemical space of SERCA inhibitors.

The machine learning approach combined with the augmented dataset of SERCA effectors reported here predicted SERCA is a pharmacological target for the anti-inflammatory drug hydrocortisone, the antibiotic penicillin V, the antifungal drug miconazole, and the Ca^2+^ channel blockers amlodipine and nimodipine. Interestingly, previous studies have shown that miconazole and amlodipine are both inhibitors of SERCA (31, 32). Additionally, our model also predicted that FDA-approved statins are inhibitors of SERCA. Statins are drugs used to treat hypercholesterolemia by inhibiting the enzyme HMG-CoA reductase, which is a rate-limiting step in the biosynthesis of cholesterol (33). Previous studies have suggested that statins stimulate SERCA activity in skeletal and vascular muscle (18, 19), in contrast with the probabilistic prediction by our model. In contrast to these studies, ATPase activation assays using isoforms SERCA1a and SERCA2a showed that most FDA-approved statins are partial inhibitors of SERCA, in agreement with the machine learning predictions. It is important to note that the machine learning model only takes into consideration the chemical characteristics of known inhibitors, several of which are partial inhibitors of SERCA. Therefore, it is expected that some classes of inhibitors with these characteristics will emerge from our machine learning-based screening, as demonstrated by the discovery of statins as partial inhibitors of SERCA.

We used complementary atomistic simulations to predict the most likely binding sites and mechanisms for inhibition (or lack thereof) of the statins discovered using our machine learning-based screening and tested experimentally. Several mechanistic insights emerged from these studies. First, extensive docking simulations, validated against crystal structures (see Supporting Information), predicted that statins bind to two known allosteric sites on SERCA, namely the TG- and CPA-binding sites. Statins bind differentially to these sites based on their functional groups: naphthalene ring-containing statins (*e.g.*, simvastatin) are predicted to bind to the transmembrane TG-binding sites, whereas the pyrrole/indole-containing statins (*e.g.*, atorvastatin) are predicted to interact with the CPA-binding site. Second, the simulations showed that pravastatin, which the model predicts as an inhibitor, but the ATPase assays show is inactive, could theoretically interact with the SERCA because it shares the chemical structure with inhibitors simvastatin and lovastatin. The atomistic simulations of the statins–SERCA complex show that compared to simvastatin and lovastatin, the naphthalene ring of pravastatin does not extend as deeply into the membrane. This positional difference, which is likely induced by the presence of the additional hydroxyl group, may explain the change in efficacy observed in the ATPase activity assays. Therefore, the simulations help explain the apparent contradiction between the prediction by the machine learning model and the inactivity of pravastatin observed experimentally.

We found that the activity of atorvastatin, the most potent statin from our screening, correlates with the formation of a stable, energetically favorable binding of this molecule with the CPA-binding site. We note some limitations of the simulations; for example, we found differences in the inhibitory activity of some statins (*e.g.*, fluvastatin) on SERCA1a and SERCA2a. The binding sites for these statins are highly conserved and therefore the differences cannot be explained by the simulations alone. Despite these limitations, the simulations provide important mechanistic insights into SERCA inhibition by statins and provide functional hypotheses that can be further tested through SERCA mutagenesis studies.

### Relationship to previous studies

In the clinical setting, lovastatin and simvastatin are administered in a pharmacologically inactive lactone form and converted to an active acid form in the body. In contrast, statins atorvastatin, fluvastatin, pitavastatin, pravastatin, and rosuvastatin are administered in the active acid form. We found that the lactone form is generally more inhibitory against SERCA than the active acid form of statins. While this suggests that statins will be predominantly found in the less inhibitory acid form, the metabolism of statins is complex, involving acid/lactone interconversion by various pathways, including glucuronidation and elimination (34, 35). As a result, both chemical forms of these statins are detected in human plasma after administration (36). This evidence suggests that SERCA inhibition by the lactone form may be possible in certain situations, *e.g.*, long-term statin therapy, thus providing a mechanistic explanation for statin toxicity (13) that increases with rising plasma concentrations of statins (37). We also found that all statins tested here are partial inhibitors of SERCA. It can be argued that partial inhibition may not be sufficient to trigger SERCA-dependent functional effects. However, previous studies have shown that this is not the case. Indeed, CXL017, a small molecule developed for the treatment of multidrug-resistant leukemia (38, 39) elicits its cytotoxic effect through partial inhibition of SERCA (40).

The effects of statins on intracellular Ca^2+^ dynamics are complex and appear to involve the inhibition of L-type Ca^2+^ channels (41), the ryanodine receptor (42, 43) and SERCA (this study). There is evidence that atorvastatin prevents Ca^2+^ uptake by SR and non-SR Ca^2+^ stores (44), in agreement with our findings showing that atorvastatin is a potent partial inhibition of both skeletal and cardiac muscle SERCA. Muscle toxicity related to statins differs between dose, concentration, and statin class (37, 45, 46). Studies in patients had shown a higher percentage of patients with SAMS are treated with simvastatin, atorvastatin, and lovastatin than patients treated with pravastatin (47). The decreased toxicity of pravastatin correlates with its inability to inhibit SERCA, therefore resulting in a lower risk of SAMS (13). *In vitro* studies suggested higher cytotoxicity levels for simvastatin and atorvastatin (48), which correlates with the inhibitory effect of these statins on SERCA. Ca^2+^ dysregulation plays a central role in diabetes pathogenesis, where downregulation of SERCA function in the pancreas is central to the pathogenesis of diabetes (49). In this context, studies have shown that a high dose of atorvastatin is associated with an increased risk of baseline fasting glucose and metabolic syndrome features (50), whereas pravastatin has shown the lowest risk of diabetes mellitus associated with statin use (51). While these side effects are far outweighed by the beneficial effects of statins on cardiovascular disease (13), our study demonstrates the applicability of data augmentation and machine learning-based screening for the identification of off-target pathways and molecular mechanisms for approved and investigational drugs.

## Experimental procedures

### Data preprocessing

For the initial training of the model, we used a dataset of 68 SERCA inhibitors and 37 inactive compounds identified by virtual screening and validated experimentally (52). All compounds’ simplified molecular-input line-entry system (SMILES) were verified and transformed to canonical SMILES using DataWarrior open software (https://openmolecules.org/datawarrior/). SERCA inhibitors activity values were discretized, labeling one for active compounds (inhibitors) and 0 for inactive compounds. To further increase the molecular diversity of putative SERCA effectors, we generated decoys using the DeepCopy generator (53). Canonical SMILES were input to the Chem library from RDKit to calculate chemical Molecular access system keys (54) and the extended connectivity radius of two fingerprints. All representations were concatenated with seven molecular and physicochemical properties of each molecule (LogP, hydrogen bond donor and acceptors, molecular weight, topological polar surface area, rotatable bonds, and the number of rings), and standardized using the Z-score normalization using StandardScaler in Scikit-learn as used earlier by us (5). We generated a second augmented training dataset of SERCA inhibitors using the CReM package (55). Briefly, we used the grow function with a conservative radius of three and the CHEMBL fragment dataset. We generated 20 molecules per compound (*i.e.*, 1360 inhibitors and 740 inactive molecules), which were included in the training set labeled as the source molecule. The initial and augmented datasets of SERCA inhibitors were subjected to stratified splitting as 85% for the training set and 25% for the testing set with a constant random state.

### Machine learning model training

To choose the best model and molecular representation for the classification problem, we compared the accuracy, receiver-operator curve area under the curve, and F1 score of 30 different classification algorithms using the Lazypredict_Supervised module included in the Lazy Predict library. The selected model and molecular representation, Random Forest with balance class weight and the concatenate extended connectivity fingerprints *n* = 2 and physiochemical properties, was fed with the preprocessed label data for a grid search of hyperparameters and cross-validation (5CV for the original set and 10CV for the augmented set) using recall as a scoring function. The model performance in the training and test sets was evaluated using several metrics as described by us recently, including receiver-operator curve area under the curve, accuracy, Matthews correlation coefficient, and F1 score (5).

### Screening and hit selection

The model trained with the augmented training dataset was used here to probe the map of SERCA inhibition using 1547 FDA-approved drugs. Briefly, we performed a dimensionality reduction and visualization for the FDA-approved molecules using t-SNE (16) on molecules PathFp descriptors in DataWarrior (56). We selected molecules using a model classification probability larger than 0.6 and neighbor count equal to or larger than three from a similarity network as initial criteria for hit filtering. We performed additional filtering using common molecular and physicochemical properties values, including hydrogen bond acceptors between 0 and 10, hydrogen bond donors between 0 and 5, cLogP between 0 and 5, and a molecular weight between 300 and 1000 g/mol.

### Chemicals

All chemicals used in this study were purchased at reagent quality (purity >95% by HPLC): atorvastatin hydroxyglutaric acid calcium salt (USP), fluvastatin hydroxyglutaric acid sodium salt (USP), pitavastatin hydroxyglutaric acid calcium salt (Sigma), rosuvastatin hydroxyglutaric acid calcium salt (Sigma), lovastatin hydroxyglutaric acid sodium salt (Merk & Co), simvastatin hydroxyglutaric acid sodium salt (Merk & Co), pravastatin hydroxyglutaric acid sodium salt (Sigma), atorvastatin lactone (Santa Cruz Biotechnology Inc), fluvastatin lactone (Santa Cruz Biotechnology Inc), pitavastatin lactone (Cayman Chem), rosuvastatin lactone (Cayman Chem), lovastatin lactone (MCE), simvastatin lactone (MCE), pravastatin lactone (Cayman Chem), and TG (Sigma).

### Isolation of enriched SERCA1a microsomes

Rabbit fast-twitch muscles were collected immediately after euthanasia and kept in ice until processing. Approximately 50 grams of muscle were minced and blended in 150 ml of a buffer containing 100 mM KCl 100, 2.5 mM K_2_HPO_4_, 2.5 KH_2_PO_4_, and 2 mM EDTA (pH = 7.4), and a protease inhibitor cocktail (Sigma). The mixture was centrifuged at 6400*g* for 20 min at 4 °C to remove a major part of the extracellular connective tissue. The obtained supernatant was filtered and centrifuged at 9700*g* for 20 min at 4 °C to remove the final remnants of cell debris. The filtrate was collected and centrifuged at 47,800*g* for 60 min at 4 °C; the pellet was resuspended and homogenized using a Teflon homogenizer in 150 ml of a solution containing 1 M sucrose and 50 mM KCl. The suspension was centrifuged at 4300*g* for 30 min at 4 °C. The supernatant obtained was mixed and incubated for 1 h with a buffer containing 2 M KCl and 100 mM Mg^2+^-ATP. The preparation was centrifuged at 84,500*g* for 90 min at 4 °C and the pellet was resuspended and homogenized in a 50 mM KCl solution. The suspension was centrifuged at 84,500*g* for 90 min at 4 °C, and the pellet was resuspended in a solution that contained 5 mM Hepes, and 300 mM sucrose. The protein concentration of microsomes was determined with a Pierce Coomassie plus assay kit (Thermo Fisher Scientific). The microsomes were aliquoted, flash-frozen in liquid nitrogen, and stored at −80 °C until use.

### Isolation of enriched SERCA2a microsomes

Pig hearts were obtained after euthanasia and placed in a cardioplegic solution (280 mM glucose, 13.44 mM KCl, 12.6 mM NaHCO_3_, and 34 mM mannitol). Left ventricles free walls were obtained, minced, and homogenized with a cold buffer that contained 9.1 mM NaHCO_3_, 0.9 mM Na_2_CO_3,_ and a cocktail of proteases inhibitors (Sigma); the mixture was centrifuged at 6500*g* for 30 min at 4 °C to remove debris. The supernatant was filtered, collected, and centrifuged at 14,000*g* for 30 min at 4 °C. The collected filtrate was centrifuged at 47,000*g* for 60 min at 4 °C. The pellet was resuspended in a solution containing 0.6 M KCl and 20 mM Tris (pH = 6.8). The suspension was centrifuged at 120,000*g* for 60 min at 4 °C and the pellet was resuspended in a solution containing 0.3 M sucrose, 5 mM 3-(N-morpholino)propanesulfonic acid, and protease inhibitors (pH = 7.4). The protein concentration of the SR microsomal fraction was determined using the PierceTM Coomassie plus assay kit (Thermo Fisher Scientific). The microsomal membranes were aliquoted, flash-frozen in liquid nitrogen, and stored at −80 °C.

### SERCA ATPase activity assays

We performed SERCA1a and SERCA2a activity assays using an enzyme-coupled NADH-linked ATPase assay as described (57, 58), with some modifications. We measured the activity of Ca^2+^ ATPase in μmol/min/mg from the decrease in absorbance of NADH at 340 nm at 25 °C in a 96-well format using an H1 Synergy (BioTek) microplate reader. Each well contained a 200 μl final volume of assay buffer containing SERCA buffer (50 mM MOPS, 100 mM KCl, 5 mM MgCl_2_, and 1 mM EGTA, pH = 7), 5 U lactate dehydrogenase, 5 U pyruvate dehydrogenase, 1 mM phosphoenolpyruvate, 5 mM ATP, 0.2 mM NADH, 2 μg of microsomal suspension, 2 μM of Ca^2+^ ionophore A23187, and a free Ca^2+^ concentration of 2.5 μM. Each concentration of the compounds tested here was calculated to a final volume of 200 μl. All compounds were incubated for 30 min at 25 °C with the reaction mixture. Concentration-response curves were constructed using a nonlinear regression fit at compound concentrations of 0.1, 0.25, 0.5, 1, 2.5, 5, 10, 25, 50, and 100 μM. Each plate included untreated microsomes as a negative control as well as microsomes treated with the SERCA inhibitor TG as a positive control. Data were fit to an inhibitor concentration *versus* normalized response curve. When applicable, IC_50_ values were calculated from the fitted concentration-response curve using the equation Y = Min + [(Max-Min)/(1 + (IC50/X)∧Hill Slope)]. Data are reported as average ± SD (N = 3). Data analysis was performed using GraphPad Prism 9.3.1 (www.graphpad.com).

### Statins setup for molecular simulations

The 3-dimensional structures of the lactone and acid forms of statins: lovastatin (CIDs: 53232 and 16760544), simvastatin (CIDs: 54454 and 10961424), pravastatin (CIDs: 9931182 and 54687), fluvastatin (CIDs: 29980473 and 446155), atorvastatin (CIDs: 6483036 and 60823), rosuvastatin (CIDs: 29918986 and 446157), and pitavastatin (CIDs: 9801294 and 5282452) were retrieved from the PubChem database (14). The structure of SERCA inhibitors was obtained directly from the crystal structures as follows: 2,5-di-*tert*-butylhydroquinone (Protein Data Bank [PDB]: 2agv (59)), biselyngbyolide B (PDB: 4ycn (60)), biselyngbyaside (PDB: 4ycm (60)), α-CPA (PDB: 2o9j (61)), TG (PDB: 1iwo (62)), and a tetrahydrocarbazole derivative (THC-7, PDB: 5ncq (63)). All compounds were submitted to geometry optimization and energy minimization using the MMFF94s force field using in the Avogadro package (64). Passive diffusion profiles of statins and TG across a 1,2-dioleoyl-sn-glycero-3-phosphocholine black lipid bilayer at 300 K and pH 7.1 were computed using the *Drag* method using the PerMM server (65).

### Ensemble docking simulations

We used 74 SERCA structures reported in the literature and curated in a previous study (6); additionally, we followed the same setup protocol for 11 SERCA structures recently deposited in the PDB (66). Therefore, 85 SERCA structures were used in this study. Prior to the docking studies, we tested both AutoDock Vina 1.2 (67) and AutoDock4 (https://autodock.scripps.edu/) scoring functions (68) to determine the molecular docking protocol that best reproduces the binding modes of the six inhibitors cocrystallized with SERCA; see Validation of the molecular docking scoring function section and Table S1 in the Supporting Information. Based on these extensive validation studies, we chose AutoDock Vina 1.2 (https://vina.scripps.edu/) scoring function for the docking studies described in this study. Statins were docked into known inhibitory sites of SERCA using 85 SERCA structures and AutoDock Vina version 1.2.1 (67). We docked the molecules on three sites: (i) the canonical sarcolipin/phospholamban site, (ii) the TG-binding site, and (iii) the CPA-binding site. We performed a total of 1190 independent docking calculations. Docking was performed within a 30 × 30 × 30 Å grid box with an exhaustiveness value of 50 and selecting the 20 best poses for further analysis.

### MD simulations

The SERCA–statin complexes obtained from the ensemble docking study were inserted in a pre-equilibrated bilayer containing a total 448 molecules of 1-palmitoyl-2-oleoyl-sn-glycero-3-phosphocholine lipids employing the *membrane builder* module of CHARMM-GUI web server (69, 70). We solvated each system using the TIP3P model of water with a periodic box with a minimum margin of 20 Å between the protein and the *z*-axis edges. Na^+^ and Cl^−^ ions were randomly added to neutralize the total charge of the systems and reach a concentration of ∼0.15 M. The systems were prepared, energy minimized, and equilibrated following the six-step setup protocol implemented by CHARMM-GUI: two 25-ps restrained canonical ensemble (NVT) simulations, one 25-ps restrained isothermal-isobaric ensemble (NPT) simulations, and three 100-ps restrained NPT simulations. The Langevin thermostat was used to keep the temperature at 310 K and the Monte Carlo barostat to maintain a constant pressure of 1.0 bar. Bonds involving hydrogen atoms were constrained using the SHAKE algorithm. We performed 200-nsMD simulations of the complexes using AMBER20 on Tesla V100 GPUs (71) and the AMBER ff14SB force field (72).

### Analysis of the MD trajectories and energy calculations

All SERCA-statin trajectories and coordinates were converted to GROMACS-type files. The tilt angles of lovastatin, pravastatin, and simvastatin relative to the membrane normal were computed using *helanal* modules of MDAnalysis. RMSD of the statins’ heavy atoms after protein backbone least squares fit was calculated with the *rms* GROMACS built-in tool. The RMSD matrix was subsequently employed to perform the clustering analysis with the *gromos* method. The *g_mmpbsa* package (73) was employed to calculate the binding enthalpy (ΔH_bind_) of the statins at the proposed binding sites in SERCA1a. For the ligands found at the TG site, we modified the solvent dielectric constant for the polar solvation calculation using the Adaptive Poisson–Boltzman Solver (74). We determined the occupancy fraction of the hydrophobic and hydrogen bonds interactions of the statins with SERCA from all the simulation snapshots with PLIP (75).

## Data availability

Data are available upon request. Contact L. Michel Espinoza-Fonseca, Center for Arrhythmia Research, NCRC Building 26, University of Michigan, Ann Arbor, MI 48109 (lmef@umich.edu).

## Supporting information

This article contains supporting information (6, 61, 76, 77, 78).

## Conflict of interest

The authors declare that they have no known competing financial interests or personal relationships that could have appeared to influence the work reported in this paper.
