# Supplementary material for: A novel machine learning-based screening identifies statins as inhibitors of the calcium pump SERCA
Source: J Biol Chem. 2023 Apr 6;299(5):104681. doi: 10.1016/j.jbc.2023.104681 (PMC10193016; doi:10.1016/j.jbc.2023.104681)
Supplement: Supporting Figures S1–S5 and Tables S1–S5 [file mmc1.pdf]

Supporting Information

A novel machine learning-based screening identifies statins as inhibitors of the calcium pump SERCA

Carlos Cruz-Cortés,[1,‡] M. Andrés Velasco-Saavedra,[1,2, ‡] Eli Fernández-de Gortari,[3, ‡] Guadalupe Guerrero-Serna,[1] Rodrigo Aguayo-Ortiz,[2] and L. Michel Espinoza-Fonseca[1,*]

[1]Center for Arrhythmia Research, Department of Internal Medicine, Division of Cardiovascular Medicine, University of Michigan, Ann Arbor, MI 48109, USA; [2]Universidad Nacional Autónoma de México, Mexico City, 04510, Mexico. [3]International Iberian Nanotechnology Laboratory, Braga, 4715-330, Portugal.

Validation of the molecular docking scoring function to model small molecule–SERCA interactions

We used molecular docking and all-atom simulations as a complementary approach to ATPase assays to study at atomic-level detail the interactions of SERCA with drug hits and to mechanistically explain our experimental results. Crystallographic data guide these modeling studies because our machine learning model was trained using inhibitors binding to sites elucidated by x-ray crystallography. Small molecules and regulatory peptides that inhibit pump activity have been found predominantly in three sites: The regulatory (canonical) binding site that binds SERCA regulators phospholamban and sarcolipin (BS1), the thapsigargin-binding site (BS2), and the "doorstop" binding site, where inhibitor CPA and BHQ interacts with the pump (BS3) (**See Chart 1**). BS1 and BS2 sites are primarily hydrophobic grooves embedded in the membrane, whereas BS3 is a relatively small pocket near the cytosolic side of the TM domain.[1]

We used both AutoDock vina (ADv 1.2) and AutoDock v4 (AD4) scoring functions to determine the molecular docking protocol that best reproduces the binding modes of the six inhibitors co-crystallized with SERCA. For the AD4 scoring, the solvent dielectric constant was set to a value of 20 to mimic the hydrophobic conditions of the membrane interfacial region.[2] Analysis of both the ranking function (pose) and the root-mean-square deviation (RMSD) values computed with DockRMSD[3] between the co-crystalized ligands and the best-scored docked poses showed that the ADv 1.2 scoring function outperforms the AD4 scoring. Indeed, we found that the top-ranking poses predicted by the ADv 1.2 scoring function closely match the geometry of those found in the crystal structures (e.g., RMSD value of ~1 Å for TG, Table 2). We also found that while the ADv 1.2 scoring function reproduces the crystallographic poses of most inhibitors tested here, its performance is modest when applied to the inhibitor CPA (RMSD =3.7 Å). This finding is not surprising considering that CPA binds to SERCA in a [Mg^{2+}]-dependent manner,[4] forming a Mg^{2+} ion-coordinated CPA–SERCA complex.[5] A summary of our docking validation analysis is shown in **Table S1**. Overall, our extensive docking studies indicate that the ADv 1.2 scoring function alone is sufficiently robust to predict structures of the inhibitor-SERCA complexes. Therefore, we chose the ADv 1.2 scoring function for the docking studies described in this study.

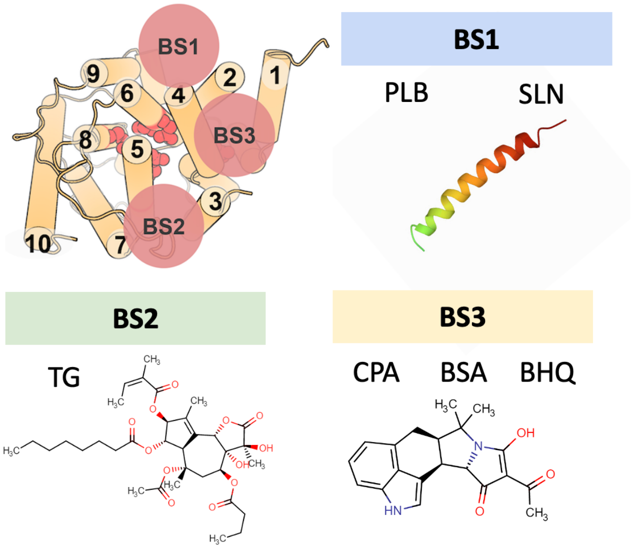

Chart 1. Localization of the regulatory binding sites BS1, BS2 and BS3 of SERCA. We show representative proteins, and small molecules that target each of these sites.

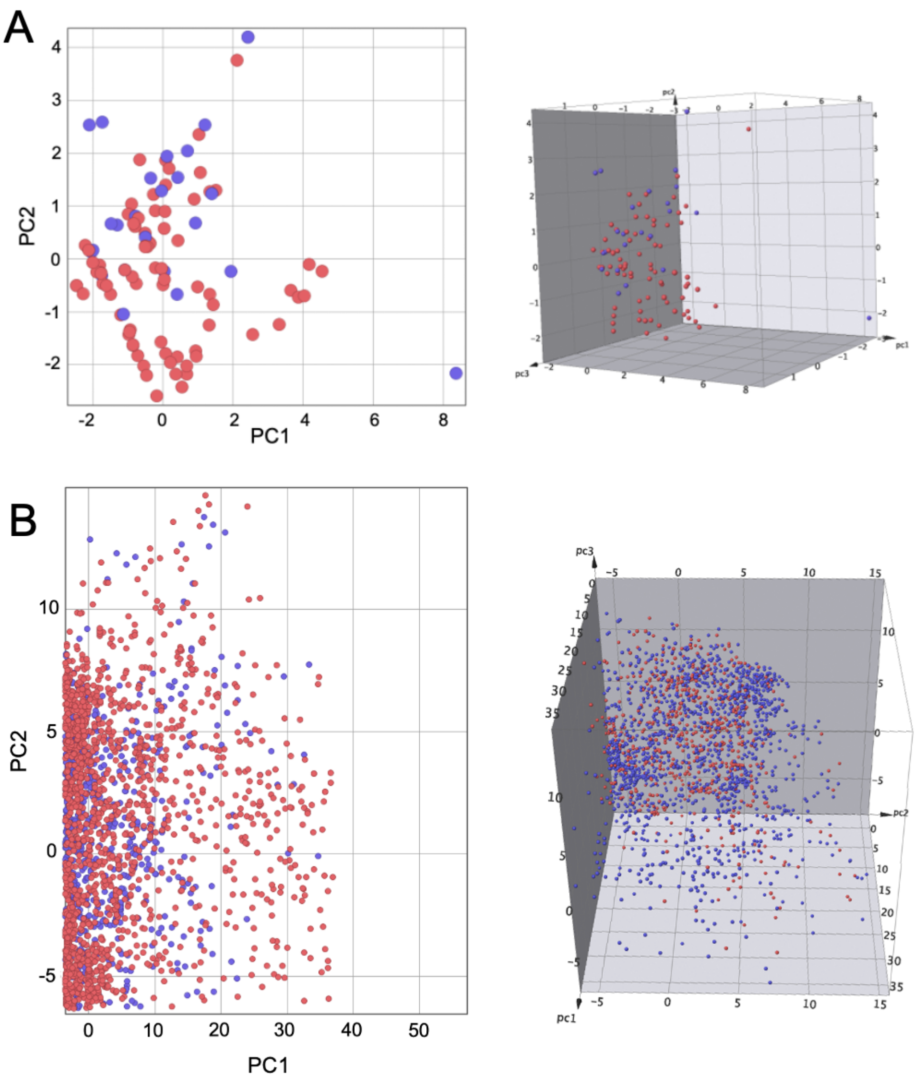

Figure S1. Original and augmented datasets of SERCA inhibitors. Two-dimensional molecular representation of the properties space by principal component analysis of the (A) original and (B) augmented datasets of SERCA effectors. The active molecules (inhibitors) are shown as red circles, whereas the inactive hits (decoys) are shown as blue circles.

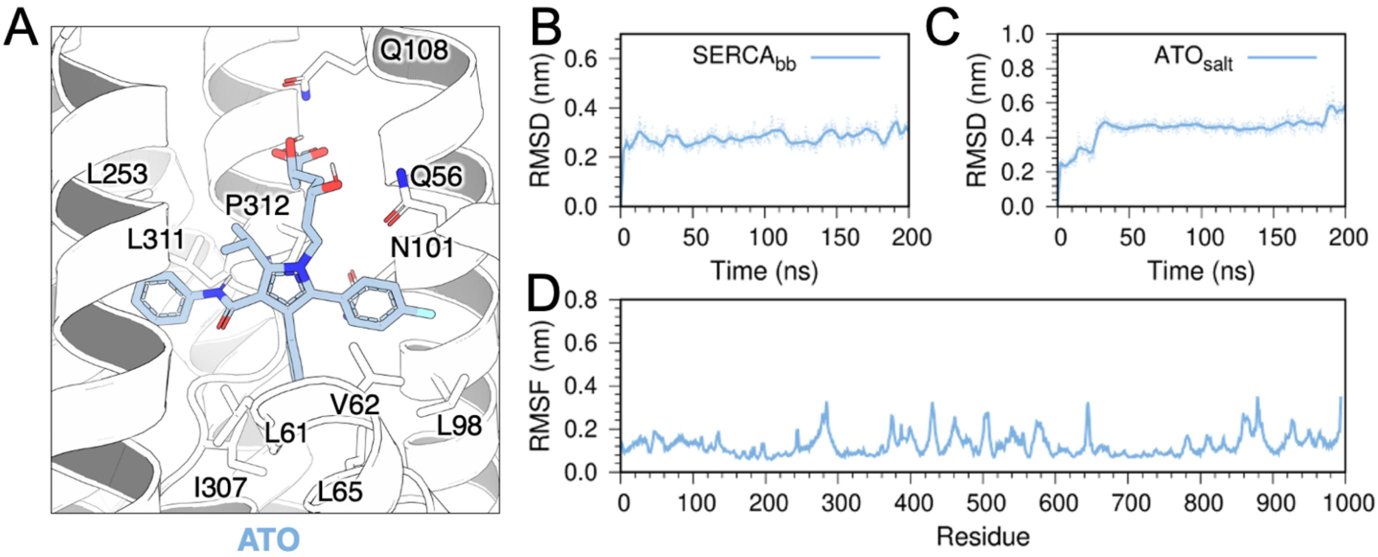

Figure S2. Docking and molecular simulation of the complex between atorvastatin hydroxyglutaric acid and SERCA. (A) A representative structure of atorvastatin hydroxyglutaric acid bound to SERCA. SERCA is shown as ribbons; interacting SERCA residues and atorvastatin are shown as sticks. (B) Root-mean square fluctuation of SERCA in the 200-ns MD simulation. (C) Root-mean square deviation of the atorvastatin hydroxyglutaric acid (salt) in the MD trajectory. (D) root-mean square fluctuation of the alpha carbons of SERCA in the MD simulation of the complex with the statin.

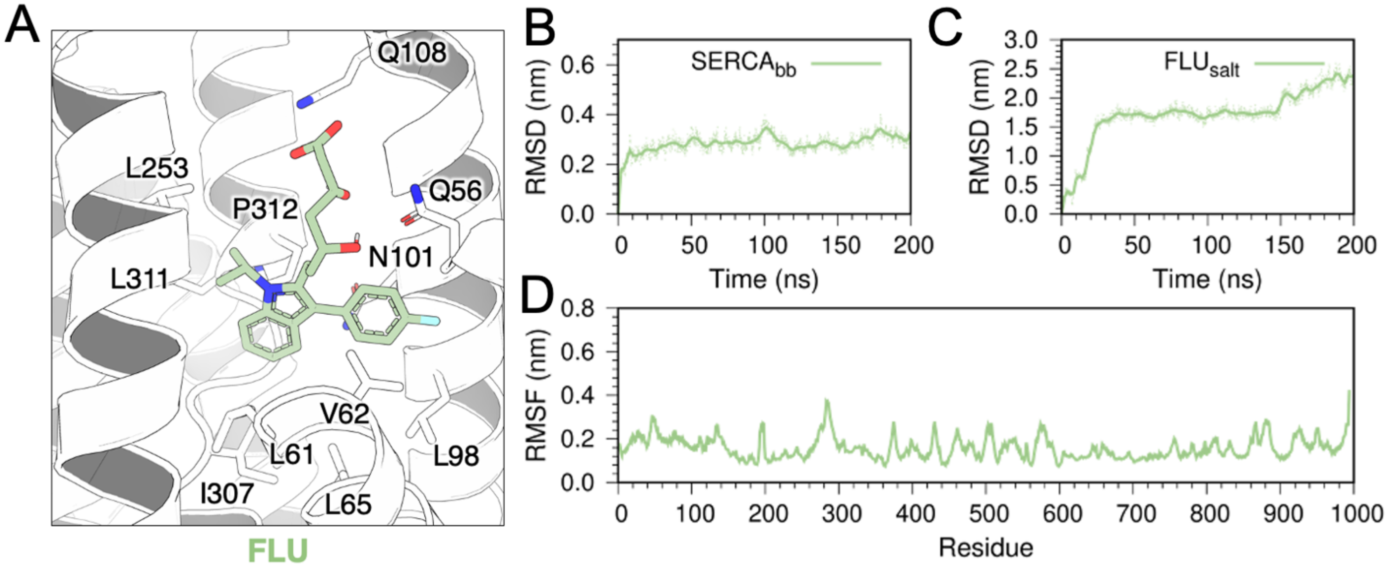

Figure S3. Docking and molecular simulation of the complex between fluvastatin hydroxyglutaric acid and SERCA. (A) A representative structure of fluvastatin hydroxyglutaric acid bound to SERCA. SERCA is shown as ribbons; interacting SERCA residues and fluvastatin are shown as sticks. (B) Root-mean square fluctuation of SERCA in the 200-ns MD simulation. (C) Root-mean square deviation of the fluvastatin hydroxyglutaric acid (salt) in the MD trajectory. (D) Root-mean square fluctuation of the alpha carbons of SERCA in the MD simulation of the complex with the statin.

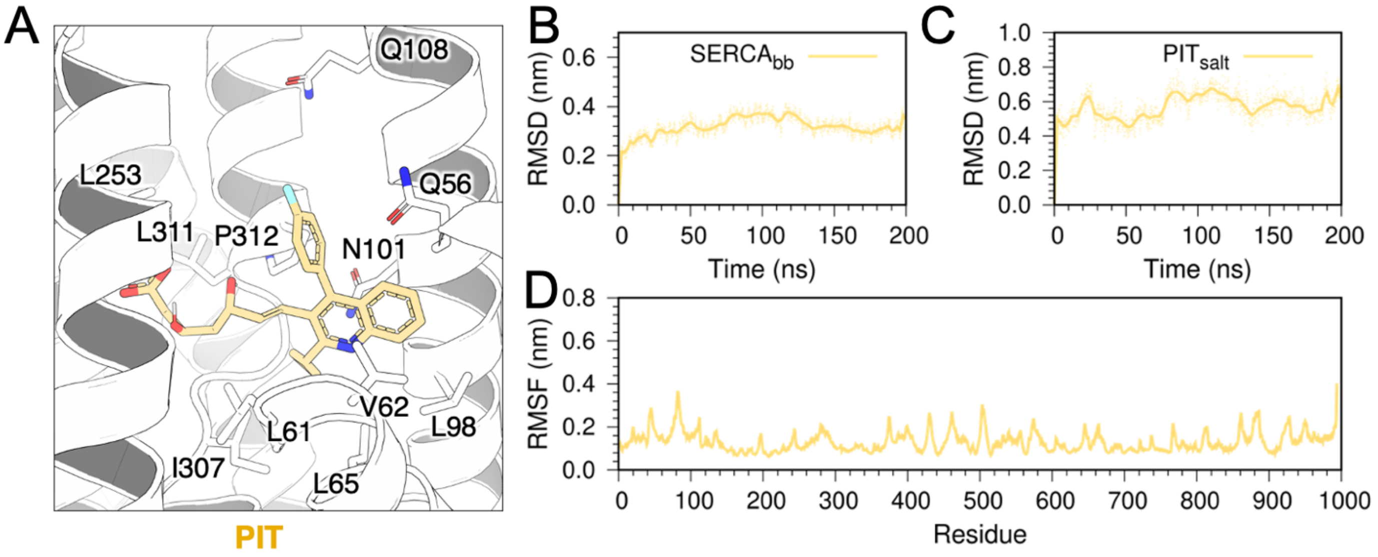

Figure S4. Docking and molecular simulation of the complex between pitavastatin hydroxyglutaric acid and SERCA. (A) A representative structure of pitavastatin hydroxyglutaric acid bound to SERCA. SERCA is shown as ribbons; interacting SERCA residues and pitavastatin are shown as sticks. (B) Root-mean square fluctuation of SERCA in the 200-ns MD simulation. (C) Root-mean square deviation of the pitavastatin hydroxyglutaric acid (salt) in the MD trajectory. (D) Root-mean square fluctuation of the alpha carbons of SERCA in the MD simulation of the complex with the statin.

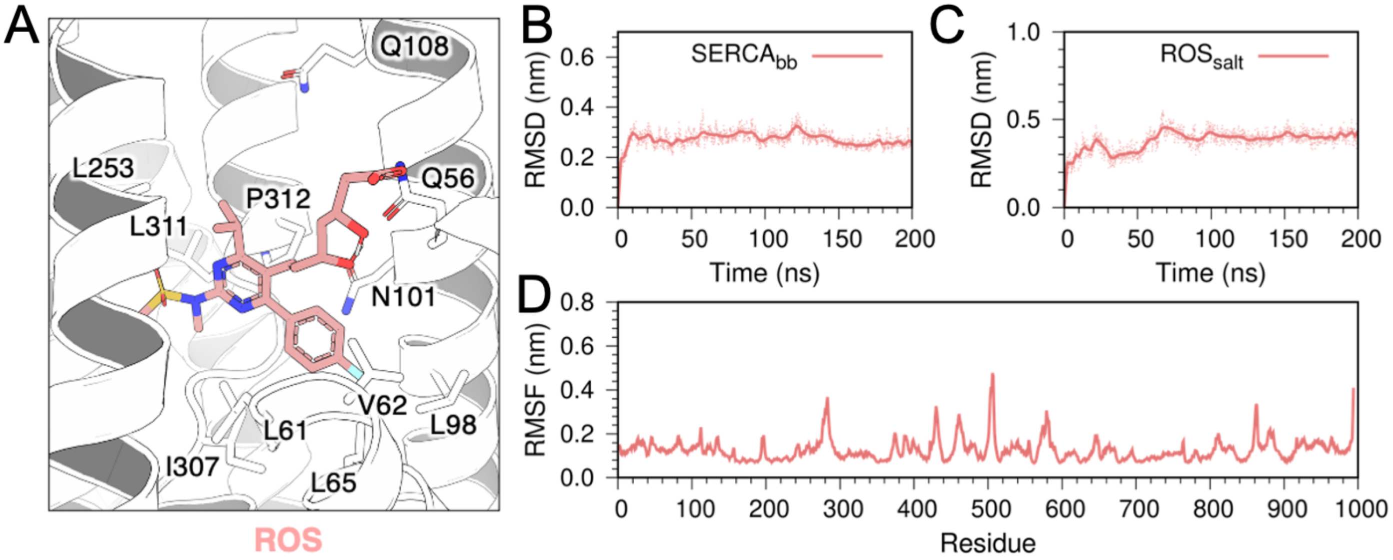

Figure S5. Docking and molecular simulation of the complex between rosuvastatin hydroxyglutaric acid and SERCA. (A) A representative structure of rosuvastatin hydroxyglutaric acid bound to SERCA. SERCA is shown as ribbons; interacting SERCA residues and rosuvastatin are shown as sticks. (B) Root-mean square fluctuation of SERCA in the 200-ns MD simulation. (C) Root-mean square deviation of the rosuvastatin hydroxyglutaric acid (salt) in the MD trajectory. (D) Root-mean square fluctuation of the alpha carbons of SERCA in the MD simulation of the complex with the statin.

Table S1. Parameters from the concentration-response fitting model calculated for inhibition of SERCA1a

Drug	Bottom	Top	IC_{50} (μM)	% Maximal inhibition	R^2
Lovastatin lactone	0.0 ± 0.55	0.97 ± 0.01	167.3 ±139.5	N/D	0.83
Lovastatin acid	0.46 ± 0.35	1.03 ± 0.01	154.7±145	N/D	0.80
Simvastatin lactone	0.0 ± 0.28	1.01 ± 0.02	106 ± 52	N/D	0.90
Simvastatin acid	0.0 ± 0.16	1.03 ± 0.01	91 ± 73	32 ± 15	0.77
Pravastatin lactone	0.96 ± 0.01	1.18 ± 1.05	N/D	N/D	0.11
Pravastatin acid	1.06 ± 0.03	1.01 ± 0.01	N/D	N/D	ND
Atorvastatin lactone	0.42 ± 0.05	0.99 ± 0.02	10.7 ± 3.4	58 ± 4	0.89
Atorvastatin acid	0.0 ± 0.5	0.98 ± 0.01	N/D	N/D	0.90
Fluvastatin lactone	0.44 ± 0.46	0.99 ± 0.01	N/D	N/D	0.65
Fluvastatin acid	0.0 ±1.3	0.99 ± 0.01	N/D	N/D	0.83
Pitavastatin lactone	0.29±0.08	0.98 ± 0.01	34.8 ±10.1	71 ± 8	0.93
Pitavastatin acid	0.0 ± 15.26	0.98 ± 0.01	N/D	N/D	0.24
Rosuvastatin lactone	0.25 ±0.20	0.97 ± 0.01	95.56 ± 46.67	75 ± 20	0.90
Rosuvastatin acid	0.0 ± 16.3	0.99 ± 0.01	N/D	N/D	0.24

Table S2. Parameters from the concentration-response fitting model calculated for inhibition of SERCA2a

Drug	Bottom	Top	IC_{50} (μM)	% Maximal inhibition	R^2
Lovastatin lactone	0.0 ± 0.32	0.95 ± 0.01	121 ± 66	N/D	0.90
Lovastatin acid	0.66 ± 0.35	1.01 ± 0.01	N/D	N/D	0.57
Simvastatin lactone	0.30 ± 0.06	1.02 ± 0.02	22.7 ± 6.1	70 ± 6	0.93
Simvastatin acid	0.71 ± 0.21	0.99 ± 0.01	N/D	N/D	0.58
Pravastatin lactone	0.95 ± 0.02	1.00 ± 0.01	N/D	N/D	0.18
Pravastatin acid	0.97 ± 0.01	0.98 ± 0.01	N/D	N/D	0.1
Atorvastatin lactone	0.51 ± 0.02	1.01 ± 0.02	3.9 ± 0.8	49 ± 2	0.95
Atorvastatin acid	0.61 ± 0.07	0.96 ± 0.01	45.9 ± 20	39 ± 7	0.87
Fluvastatin lactone	0.35 ± 1.67	0.97 ± 0.01	N/D	N/D	0.56
Fluvastatin acid	0.74 ± 0.18	0.9 ± 0.01	N/D	N/D	0.55
Pitavastatin lactone	0.17 ± 0.10	0.96 ± 0.02	38.9 ± 11.9	83 ± 10	0.93
Pitavastatin acid	0.88 ± 0.03	0.97 ± 0.01	N/D	12 ± 3	0.40
Rosuvastatin lactone	0.47 ± 0.06	1.00 ± 0.01	46.8 ± 12.3	53 ± 6	0.95
Rosuvastatin acid	N/D	N/D	N/D	N/D	0.04

Table S3. Validation of the docking model used in this study.

Ligand			Vina + AD4 score*			ADv 1.2		
Inhibitor	PDB ID	State	Score (kcal/mol)	RMSD (Å)	Pose	Score (kcal/mol)	RMSD (Å)	Pose
TG	1iwo	E_2-TG	-7.765	1.912	16	-7.713	1.075	2
BLS	4ycm	E_2	-8.654	2.681	7	-9.538	2.101	2
BLLB	4ycn	E_2	-9.106	1.414	4	-10.076	2.180	1
BHQ	2agv	E_2-TG	-6.521	0.637	1	-7.177	0.274	1
THC 7	5ncq	E_2-ATP	-11.008	0.768	3	-8.884	1.282	1
CPA	2o9j	E_2-P_i	-7.156	1.466	5	-9.496	3.679	1

*Vina + AD4 was used considering the dielectric constant as 20 instead of 80.

Table S4. Scores for statin lactones docked onto thapsigargin (BS2) and cyclopiazonic acid (BS3) binding sites using all crystal structures of SERCA available in the literature. Docking score values are in kcal·mol^{-1}.

PDB ID	State	LOV		SIM		PRA		ATO		FLU		PIT		ROS	
		BS2	BS3	BS2	BS3	BS2	BS3	BS2	BS3	BS2	BS3	BS2	BS3	BS2	BS3
1iwo	E_2-TG	-8.461	-8.172	-9.228	-8.353	-8.491	-8.442	-9.334	-8.924	-9.034	-8.556	-8.890	-8.480	-8.407	-7.602
1su4	E_1-2Ca	-7.674	-8.470	-7.470	-9.058	-8.030	-7.943	-9.238	-9.252	-9.368	-9.765	-9.472	-9.474	-7.330	-8.239
1t5s	E_1-2Ca-ATP	-6.981	-7.008	-7.300	-7.518	-7.823	-8.006	-8.354	-7.169	-8.651	-7.501	-8.758	-7.316	-7.990	-6.365
1t5t	E_1~P-2Ca-ADP	-7.042	-6.722	-7.065	-6.854	-7.108	-7.066	-8.128	-7.170	-7.932	-7.149	-8.270	-7.384	-7.362	-6.736
1vfp	E_1-2Ca-ATP	-7.752	-7.244	-7.347	-6.796	-7.112	-6.935	-8.552	-7.556	-8.687	-7.648	-8.553	-7.825	-7.442	-7.538
1wpg	E_2-P_i-2Ca-ADP-TG	-7.689	-8.467	-7.930	-8.226	-7.908	-8.194	-8.500	-10.232	-9.022	-9.599	-8.318	-9.453	-9.084	-8.953
1xp5	E_2~P-TG	-9.402	-8.797	-9.399	-9.071	-8.762	-8.366	-10.536	-10.317	-9.367	-8.955	-9.789	-9.683	-9.311	-8.732
2agv	E_2-TG	-9.039	-8.244	-8.901	-8.767	-8.429	-8.574	-9.724	-9.106	-9.391	-9.233	-9.158	-8.711	-8.289	-8.131
2by4	E_2-ATP	-9.634	-8.025	-9.143	-8.419	-8.897	-8.122	-9.341	-9.120	-9.187	-8.702	-9.281	-8.600	-8.627	-7.921
2c88	E_2-ATP-TG	-9.068	-8.918	-9.407	-9.131	-8.993	-8.859	-9.870	-9.048	-9.231	-9.732	-9.398	-9.724	-8.770	-9.263
2c8k	E_2-ATP-TG	-9.577	-9.124	-9.253	-9.205	-9.125	-8.448	-9.321	-9.570	-8.772	-9.647	-9.798	-10.096	-8.391	-9.078
2c8l	E_2-TG	-9.205	-9.111	-8.943	-9.340	-8.926	-8.924	-8.682	-9.293	-8.444	-9.485	-9.608	-9.739	-8.840	-8.432
2c9m	E_1-2Ca	-7.496	-8.506	-7.622	-8.245	-7.356	-7.859	-8.811	-9.801	-9.253	-9.297	-9.479	-9.367	-8.625	-8.704
2dqs	E_2-ATP-TG	-9.152	-8.270	-8.874	-8.297	-8.619	-8.294	-9.715	-9.033	-9.204	-8.983	-9.061	-9.310	-9.023	-8.314
2ear	E_2-TG	-7.866	-8.152	-7.640	-8.351	-7.948	-7.839	-9.325	-9.185	-8.880	-9.587	-8.511	-9.363	-9.039	-8.460
2eat	E_2-TG	-8.499	-8.534	-8.924	-8.651	-8.186	-7.973	-10.616	-9.782	-9.245	-9.071	-9.405	-9.453	-9.045	-8.107
2eau	E_2	-7.966	-8.664	-8.094	-8.913	-8.195	-8.991	-7.494	-8.988	-8.892	-9.518	-8.287	-9.361	-9.318	-10.119
2o9j	E_2-P_i	-8.250	-9.748	-7.489	-9.709	-7.533	-8.500	-7.763	-10.048	-8.706	-9.798	-8.415	-10.007	-8.349	-8.833
2oa0	E_2-ADP	-8.385	-7.782	-7.858	-8.340	-7.858	-8.357	-8.660	-8.456	-8.716	-8.542	-9.034	-8.799	-8.315	-8.055
2yfy	E_2-TG	-8.863	-8.583	-8.784	-8.954	-8.815	-8.700	-9.073	-10.278	-8.956	-9.605	-9.118	-9.398	-8.541	-9.256
2zbd	E_1~P-2Ca-ADP	-6.760	-7.058	-6.558	-7.033	-6.846	-7.447	-7.706	-6.683	-7.253	-7.584	-7.213	-7.910	-6.503	-7.172
2zbe	E_2P	-8.400	-8.480	-8.341	-8.307	-7.856	-8.456	-7.972	-9.128	-8.078	-8.848	-8.394	-8.788	-8.059	-8.325
2zbf	E_2P-TG	-8.899	-8.856	-8.574	-9.306	-8.388	-8.427	-8.875	-9.924	-9.502	-9.434	-9.100	-9.089	-8.127	-8.912
2zbg	E_2~P-TG	-8.833	-8.223	-8.988	-7.901	-8.942	-8.526	-9.373	-9.409	-9.007	-9.110	-8.864	-9.257	-9.413	-8.784
3ar2	E_1-2Ca-ATP	-6.903	-7.038	-6.984	-7.014	-7.173	-7.179	-7.323	-7.638	-7.413	-7.458	-7.304	-8.373	-6.813	-7.462
3ar3	E_2-ADP-TG	-9.056	-8.067	-8.665	-7.834	-8.552	-8.383	-9.441	-9.599	-8.770	-9.150	-8.909	-9.252	-8.105	-8.130
3ar4	E_2-ATP-TG	-8.995	-8.293	-9.178	-8.185	-8.811	-8.326	-10.179	-8.851	-8.872	-8.839	-9.108	-9.355	-8.304	-8.039
3ar5	E_2-ADP-TG	-8.760	-8.476	-8.495	-8.380	-8.723	-7.988	-9.200	-9.175	-9.241	-9.038	-8.828	-9.187	-8.112	-8.332
3ar6	E_2-ADP-TG	-8.936	-8.104	-8.944	-8.620	-8.668	-8.647	-9.698	-8.862	-8.522	-8.807	-9.611	-9.168	-8.663	-8.286
3ar7	E_2-ATP-TG	-9.684	-8.355	-9.091	-9.037	-8.910	-8.405	-9.507	-9.102	-9.108	-9.209	-9.075	-9.172	-8.583	-8.201
3ar8	E_2~P-ADP-TG	-9.696	-8.727	-8.910	-8.168	-9.149	-8.526	-9.320	-9.990	-9.828	-9.612	-9.005	-9.496	-9.182	-9.103
3ar9	E_2P-ADP-TG	-9.068	-8.951	-8.682	-9.005	-8.750	-8.570	-9.347	-10.500	-8.803	-9.329	-9.253	-9.808	-8.260	-8.498
3b9b	E_2P-Mg	-6.906	-7.896	-6.867	-6.970	-6.722	-6.611	-7.942	-7.042	-7.816	-7.482	-7.317	-6.415	-6.825	-6.272
3b9r	E_2~P-ATP	-6.958	-8.399	-7.785	-8.077	-7.410	-7.746	-7.401	-8.832	-7.550	-8.790	-7.191	-9.472	-6.949	-8.886
3ba6	E_1P-2Ca-ADP	-7.668	-7.500	-7.873	-6.771	-7.679	-6.881	-7.990	-8.428	-8.697	-8.350	-8.801	-7.380	-8.529	-7.171
3fgo	E_2-P_i-ATP	-7.630	-8.940	-7.221	-9.007	-8.058	-9.451	-8.201	-8.747	-9.301	-10.642	-8.846	-10.309	-8.124	-8.363
3fpb	E_2-P_i-ATP	-7.853	-9.081	-8.281	-8.908	-7.948	-8.739	-7.761	-10.159	-8.438	-10.582	-8.966	-9.858	-8.108	-9.973
3fps	E_2-ADP	-8.255	-8.489	-8.329	-8.702	-8.133	-9.133	-9.362	-9.164	-8.900	-9.759	-8.332	-10.236	-8.140	-8.410
3j7t	E_1-2Ca	-7.268	-7.467	-7.493	-7.924	-7.782	-7.916	-8.459	-9.115	-7.992	-9.379	-8.706	-9.363	-7.391	-8.548
3n5k	E_2~P-TG	-8.987	-8.524	-9.123	-8.462	-9.192	-8.602	-9.668	-9.442	-8.763	-9.376	-8.999	-9.785	-8.337	-7.928
3n8g	E_1-2Ca-ATP	-7.829	-7.145	-7.211	-7.477	-7.226	-7.582	-8.640	-8.313	-7.697	-7.441	-7.822	-7.589	-7.604	-6.673
3tlm	E_1-2Ca-ATP	-7.448	-7.511	-6.846	-6.551	-6.868	-6.668	-8.153	-8.179	-7.886	-7.581	-8.108	-7.587	-7.498	-6.721
3w5a	E_1-SLN	-7.580	-8.913	-6.404	-7.827	-8.027	-8.491	-7.378	-8.573	-6.621	-10.283	-8.389	-9.063	-6.552	-9.567
3w5b	E_1-Mg-ATP	-6.103	-7.474	-6.142	-7.619	-5.954	-7.655	-7.154	-9.538	-6.635	-8.789	-6.756	-8.522	-6.507	-8.327
3w5c	E_2	-8.154	-8.416	-8.098	-8.096	-8.390	-8.039	-9.200	-9.284	-8.537	-9.039	-8.562	-8.957	-8.301	-8.315
3w5d	E_2	-8.057	-8.319	-8.099	-7.941	-8.577	-8.087	-9.186	-9.525	-8.327	-8.974	-8.999	-9.306	-7.759	-8.106
4bew	E_2-P_i-ATP	-7.992	-9.154	-7.409	-9.776	-7.611	-8.967	-8.707	-8.879	-8.876	-10.602	-9.590	-11.386	-8.504	-8.583
4h1w	E_1-SLN	-6.839	-7.677	-6.645	-7.193	-6.379	-7.844	-6.916	-8.532	-6.679	-8.759	-7.074	-8.105	-7.458	-8.144
4j2t	E_2-TG	-9.237	-8.326	-8.837	-8.102	-9.036	-7.926	-9.665	-8.572	-8.466	-9.088	-9.145	-9.212	-8.053	-8.258
4kyt	E_1-PLB	-6.793	-9.130	-6.445	-8.631	-6.812	-8.676	-7.790	-7.377	-7.494	-9.278	-8.433	-10.339	-6.995	-9.433
4uu0	E_2-TG	-10.167	-8.771	-8.962	-8.776	-9.391	-8.610	-9.275	-8.821	-8.917	-9.191	-9.286	-8.894	-8.336	-8.909
4uu1	E_2-ATP-TG	-8.804	-8.284	-8.859	-8.509	-8.808	-8.427	-9.090	-8.889	-8.647	-9.413	-8.533	-9.379	-7.907	-8.279
4xou	E_1-2Ca-ATP	-7.206	-7.040	-7.698	-7.696	-6.990	-6.691	-7.822	-7.705	-8.279	-7.819	-8.058	-7.256	-7.428	-7.185
4y3u	E_1-PLB	-7.236	-8.446	-6.407	-8.248	-6.853	-8.515	-7.537	-7.994	-7.263	-9.140	-7.746	-9.553	-6.513	-9.095
4ycl	E_2	-9.181	-8.910	-9.080	-9.105	-8.151	-8.578	-8.338	-9.466	-9.079	-9.686	-8.347	-9.740	-8.193	-9.497
4ycm	E_2	-7.856	-8.829	-8.029	-9.215	-8.313	-9.264	-8.668	-9.677	-8.818	-9.800	-9.007	-10.569	-7.451	-8.579
4ycn	E_2	-7.030	-8.763	-6.842	-8.200	-6.932	-9.131	-8.045	-9.238	-8.171	-9.864	-7.882	-10.676	-7.013	-8.939
5a3q	E_2~P-ATP-TG	-8.567	-8.298	-8.611	-8.398	-8.400	-7.849	-9.240	-9.359	-8.621	-8.662	-9.554	-8.832	-8.754	-8.296
5a3r	E_2P-Mg-ATP	-7.678	-6.450	-7.657	-6.459	-7.760	-6.561	-8.121	-7.069	-7.716	-7.844	-8.307	-6.698	-7.494	-6.874
5a3s	E_2~P-ATP-TG	-8.711	-8.700	-8.513	-8.525	-8.631	-7.982	-9.155	-10.114	-8.959	-9.523	-9.575	-9.146	-8.026	-8.259
5mpm	E_2-P_i	-8.527	-8.767	-7.111	-9.147	-7.407	-9.313	-8.404	-12.411	-8.050	-10.807	-8.271	-11.358	-7.441	-9.695
5ncq	E_2-ATP	-8.017	-7.942	-7.280	-8.410	-7.894	-7.916	-8.548	-9.692	-9.213	-9.165	-8.929	-9.379	-8.091	-8.688
5xa7	E_1-2Ca	-7.783	-8.537	-7.788	-8.189	-7.044	-7.901	-8.630	-8.832	-8.838	-8.697	-8.873	-9.313	-8.101	-8.067
5xa8	E_1~P-2Ca-ADP	-7.033	-6.976	-7.086	-7.798	-6.366	-7.116	-7.840	-7.250	-7.560	-7.051	-8.001	-7.509	-7.017	-6.800
5xa9	E_2~P-TG	-8.860	-8.137	-8.970	-8.551	-8.895	-8.217	-9.076	-10.063	-9.501	-8.876	-9.081	-9.271	-8.612	-8.333
5xaa	E_2~P-TG	-8.765	-8.876	-8.841	-8.470	-9.098	-8.459	-9.635	-9.778	-9.424	-9.252	-9.354	-9.519	-8.550	-8.388
5xab	E_2-TG	-9.030	-8.276	-8.745	-8.711	-9.162	-8.420	-8.992	-9.116	-8.622	-8.813	-8.832	-9.637	-8.173	-8.173
5zmv	E_2-TG	-8.748	-8.567	-8.538	-8.123	-9.080	-8.820	-9.023	-9.738	-8.879	-9.161	-9.443	-8.439	-8.341	-7.928
5zmw	E_2-TG	-9.030	-8.670	-9.728	-9.387	-8.773	-8.836	-9.800	-8.687	-9.565	-8.813	-9.797	-8.812	-10.115	-7.521
5ztf	E_1-2Ca-ATP	-7.658	-7.588	-7.506	-7.866	-6.818	-6.941	-8.985	-7.911	-8.698	-8.528	-7.797	-8.024	-7.285	-7.093
6hef	E_1-2Ca-ATP	-7.250	-6.758	-7.604	-7.261	-6.786	-6.755	-8.474	-8.313	-7.712	-7.561	-7.938	-7.400	-7.621	-6.583
6hxb	E_1-2Ca-ATP	-7.238	-7.219	-7.164	-7.049	-7.269	-7.233	-8.225	-7.867	-8.025	-8.079	-7.702	-7.561	-7.164	-7.301
6jju	E_1-2Ca-ATP	-7.420	-6.949	-8.029	-7.906	-7.545	-6.570	-8.231	-7.820	-8.721	-7.658	-8.518	-7.230	-7.778	-6.683
6lle	E_1-2Ca-ATP	-6.845	-7.232	-7.067	-7.166	-6.350	-6.947	-8.089	-7.876	-8.107	-7.063	-8.236	-7.255	-6.939	-6.610
6lly	E_2P	-6.398	-6.863	-6.987	-7.451	-6.228	-6.972	-7.497	-7.397	-7.321	-7.881	-7.494	-7.814	-6.855	-7.314
6ln5	E_1-2Ca-ATP	-6.629	-6.921	-6.563	-6.454	-7.287	-6.504	-7.887	-6.687	-7.968	-7.638	-8.000	-6.800	-7.518	-6.574
6ln6	E_1-2Ca-ATP	-6.613	-6.793	-7.312	-6.696	-6.527	-6.486	-8.275	-6.753	-8.292	-7.457	-8.065	-6.908	-7.393	-6.277
6ln7	E_1-2Ca-ATP	-7.276	-6.567	-7.352	-6.521	-7.036	-6.312	-7.930	-7.035	-7.743	-7.248	-8.576	-7.326	-7.240	-6.274
6ln8	E_2P	-7.332	-6.901	-7.214	-6.891	-7.088	-6.933	-6.920	-7.669	-7.469	-7.761	-7.584	-7.636	-6.535	-7.001
6ln9	E_2P	-7.003	-8.922	-6.688	-8.628	-7.101	-8.229	-8.431	-9.072	-8.587	-9.585	-7.840	-9.118	-7.789	-8.952
6rb2	E_1-2Ca-ATP	-7.898	-7.276	-7.872	-7.640	-7.114	-7.291	-8.198	-7.391	-8.726	-7.742	-8.564	-7.627	-7.630	-6.537
6yaa	E_2-ATP	-7.685	-8.004	-7.981	-8.271	-7.728	-8.074	-7.973	-9.466	-8.788	-9.301	-8.674	-9.640	-7.440	-8.654
6yso	E_2~P-ATP-TG	-8.832	-7.827	-8.585	-8.243	-8.460	-8.674	-10.297	-9.181	-9.708	-8.950	-8.909	-8.468	-9.407	-7.929
7bt2	E_2-ATP	-9.183	-7.694	-8.897	-7.912	-8.645	-7.797	-7.992	-8.667	-9.028	-9.083	-8.667	-9.293	-7.385	-7.708
7e7s	E_1-2Ca	-7.217	-6.842	-6.854	-6.748	-7.596	-6.249	-8.281	-7.183	-7.756	-6.953	-7.975	-7.135	-7.580	-6.254

Table S5. Summary of predicted binding sites for each statin on SERCA

Statin	PDB ID	State	Binding site	Pose	Score (kcal/mol)
Lovastatin	4uu0	E_2-TG	BS2	1	-10.167
Simvastatin	5zmw	E_2-TG	BS2	2	-9.367
Pravastatin	4uu0	E_2-TG	BS2	1	-9.391
Atorvastatin	5mpm	E_2-P_i	BS3	1	-12.411
Fluvastatin	5mpm	E_2-P_i	BS3	8	-9.209
Pitavastatin	4bew	E_2-P_i-ATP	BS3	7	-9.366
Rosuvastatin	5mpm	E_2-P_i	BS3	17	-8.805

*PDBs from the molecules of the BS3 were co-crystallized with CPA.

Supplementary References

1. Aguayo-Ortiz, R.; Espinoza-Fonseca, L. M., Linking Biochemical and Structural States of SERCA: Achievements, Challenges, and New Opportunities. *Int J Mol Sci* **2020,** *21* (11).
2. Tian, P., Computational protein design, from single domain soluble proteins to membrane proteins. *Chem Soc Rev* **2010,** *39* (6), 2071-82.
3. Bell, E. W.; Zhang, Y., DockRMSD: an open-source tool for atom mapping and RMSD calculation of symmetric molecules through graph isomorphism. *J Cheminform* **2019,** *11* (1), 40.
4. Montigny, C.; Picard, M.; Lenoir, G.; Gauron, C.; Toyoshima, C.; Champeil, P., Inhibitors bound to Ca(2+)-free sarcoplasmic reticulum Ca(2+)-ATPase lock its transmembrane region but not necessarily its cytosolic region, revealing the flexibility of the loops connecting transmembrane and cytosolic domains. *Biochemistry* **2007,** *46* (51), 15162-74.
5. Moncoq, K.; Trieber, C. A.; Young, H. S., The molecular basis for cyclopiazonic acid inhibition of the sarcoplasmic reticulum calcium pump. *J Biol Chem* **2007,** *282* (13), 9748-9757.
